# Monocarboxylate Transporter 4 in Cancer-Associated Fibroblasts Is a Driver of Aggressiveness in Aerodigestive Tract Cancers

**DOI:** 10.3389/fonc.2022.906494

**Published:** 2022-06-22

**Authors:** Marina Domingo-Vidal, Diana Whitaker-Menezes, Mehri Mollaee, Zhao Lin, Madalina Tuluc, Nancy Philp, Jennifer M. Johnson, Tingting Zhan, Joseph Curry, Ubaldo Martinez-Outschoorn

**Affiliations:** ^1^Sidney Kimmel Cancer Center, Department of Medical Oncology, Thomas Jefferson University, Philadelphia, PA, United States; ^2^Lewis Katz School of Medicine, Department of Pathology and Laboratory Medicine, Temple University, Philadelphia, PA, United States; ^3^Sidney Kimmel Cancer Center, Department of Pathology, Anatomy and Cell Biology, Thomas Jefferson University, Philadelphia, PA, United States; ^4^Division of Biostatistics, Department of Pharmacology and Experimental Therapeutics, Thomas Jefferson University, Philadelphia, PA, United States; ^5^Sidney Kimmel Cancer Center, Department of Otolaryngology - Head and Neck Surgery, Thomas Jefferson University, Philadelphia, PA, United States

**Keywords:** aerodigestive tract cancer, non-small cell lung cancer, head and neck squamous cell carcinoma, cancer-associated fibroblast (CAF), metabolic compartmentalization, monocarboxylate transporter 4 (MCT4), tumor metabolism, glycolysis

## Abstract

The most common cancers of the aerodigestive tract (ADT) are non-small cell lung cancer (NSCLC) and head and neck squamous cell carcinoma (HNSCC). The tumor stroma plays an important role in ADT cancer development and progression, and contributes to the metabolic heterogeneity of tumors. Cancer-associated fibroblasts (CAFs) are the most abundant cell type in the tumor stroma of ADT cancers and exert pro-tumorigenic functions. Metabolically, glycolytic CAFs support the energy needs of oxidative (OXPHOS) carcinoma cells. Upregulation of the monocarboxylate transporter 4 (MCT4) and downregulation of isocitrate dehydrogenase 3α (IDH3α) are markers of glycolysis in CAFs, and upregulation of the monocarboxylate transporter 1 (MCT1) and the translocase of the outer mitochondrial membrane 20 (TOMM20) are markers of OXPHOS in carcinoma cells. It is unknown if glycolytic metabolism in CAFs is a driver of ADT cancer aggressiveness. In this study, co-cultures *in vitro* and co-injections in mice of ADT carcinoma cells with fibroblasts were used as experimental models to study the effects of fibroblasts on metabolic compartmentalization, oxidative stress, carcinoma cell proliferation and apoptosis, and overall tumor growth. Glycolytic metabolism in fibroblasts was modulated using the HIF-1α inhibitor BAY 87-2243, the antioxidant N-acetyl cysteine, and genetic depletion of MCT4. We found that ADT human tumors express markers of metabolic compartmentalization and that co-culture models of ADT cancers recapitulate human metabolic compartmentalization, have high levels of oxidative stress, and promote carcinoma cell proliferation and survival. In these models, BAY 87-2243 rescues IDH3α expression and NAC reduces MCT4 expression in fibroblasts, and these treatments decrease ADT carcinoma cell proliferation and increase cell death. Genetic depletion of fibroblast MCT4 decreases proliferation and survival of ADT carcinoma cells in co-culture. Moreover, co-injection of ADT carcinoma cells with fibroblasts lacking MCT4 reduces tumor growth and decreases the expression of markers of metabolic compartmentalization in tumors. In conclusion, metabolic compartmentalization with high expression of MCT4 in CAFs drives aggressiveness in ADT cancers.

## Introduction

Lung and head and neck cancers are the second and seventh leading cancers worldwide, respectively (1). Non-small cell lung cancer (NSCLC) comprises 85% of all lung cancer cases (2) and head and neck squamous cell carcinoma (HNSCC) accounts for over 90% of head and neck cancers (3). There are no curative therapies for these aerodigestive tract (ADT) cancers when metastatic, and patients frequently develop treatment resistance and cancer relapse. Moreover, NSCLC is the leading cause of cancer-related deaths worldwide (1). The overall survival rate for NSCLC is still very poor, around 20% at 5 years. The survival of HNSCC is better, around 65% at 5 years, however it has not improved in the last decade (4, 5). Therefore, further characterization of the drivers of aggressiveness in ADT cancers is needed to find novel treatment strategies and improve patient outcomes.

The metabolic interactions between carcinoma cells and stromal cells have a major influence in the development and progression of many human malignancies. Cancer-associated fibroblasts (CAFs) are a predominant stromal cell type that can account for more than 50% of the cells in ADT tumors (6, 7). Carcinoma cells metabolically reprogram CAFs in order to derive nutrients and sustain their growth and proliferation (8, 9). Metabolically reprogrammed CAFs adopt a glycolytic phenotype with secretion of high energy metabolites, such as lactate, which are used by carcinoma cells to fuel the tricarboxylic acid (TCA) cycle and obtain energy through oxidative phosphorylation (OXPHOS) (9–11). The metabolic symbiosis between highly glycolytic CAFs and oxidative carcinoma cells is known as two-compartment tumor metabolism (12). This metabolic compartmentalization exists in many human cancers (13–25), yet remains to be investigated in detail in ADT cancers.

Two main drivers of glycolytic reprogramming in CAFs are oxidative stress and stabilization of the hypoxia inducible factor 1-alpha (HIF-1α), which is a master regulator of glycolysis (9, 26). Reactive oxygen species (ROS) and HIF-1α drive the expression of the glucose transporter 1 (GLUT1) and the monocarboxylate transporter 4 (MCT4) which are two key rate limiting steps of glycolysis (27–30). GLUT1 mediates the uptake of glucose into cells and MCT4 mediates the extrusion of lactate, the end-product of glycolysis, to the extracellular media. MCT4 is an established marker of glycolysis in CAFs and is found upregulated in the stroma of many human cancers (14, 15, 18–20, 31). In fact, elevated stromal MCT4 has been associated with decreased overall survival in HNSCC (32), however this relationship has not yet been established in NSCLC. Moreover, whether MCT4 expression in CAFs is a key metabolic determinant of ADT cancer malignancy remains to be elucidated. Another marker of glycolytic reprogramming in CAFs is downregulation of the TCA cycle enzyme isocitrate dehydrogenase 3-alpha (IDH3α) (33). IDH3α downregulation induces HIF-1α stabilization and glycolysis in CAFs and promotes melanoma and colorectal cancer tumor growth (33). Moreover, HIF-1α has been shown to induce the mitochondrial isoform of the phosphoenolpyruvate carboxykinase (PEPCK-M) (34, 35), an enzyme that generates phosphoenolpyruvate from oxaloacetate and is involved in the metabolic adaptations to nutritional stress. Specifically, PEPCK-M coordinates the ability of carcinoma cells to alternatively uptake and utilize glucose, glutamine or lactate for production of energy or generation of biomass when availability of their primary metabolic source is limited (34, 36–38). The expression of IDH3α and PEPCK-M have not yet been studied in fibroblasts in ADT cancers.

In the carcinoma cell compartment, markers of OXPHOS metabolism are upregulation of the monocarboxylate transporter 1 (MCT1), the main importer of lactate into cells, and the translocase of the outer mitochondrial membrane 20 (TOMM20), a component of the receptor complex that imports mitochondrial proteins synthesized in the cytosol. MCT1 and TOMM20 are found highly expressed in many human cancers, including ADT cancers, and predict for aggressive disease and poor prognosis (17, 19, 39–48). The presence of glycolytic fibroblasts has been shown to upregulate these two OXPHOS markers in experimental models of HNSCC (49–52). This effect has not yet been investigated in NSCLC. Moreover, the influence of glycolytic CAFs on carcinoma cell aggressiveness and tumor growth has been poorly characterized in ADT cancers. In this study, we have determined that the presence of fibroblasts drives metabolic compartmentalization and carcinoma cell aggressiveness in ADT cancer experimental models, and that altering fibroblast glycolytic metabolism reduces ADT tumor growth.

## Materials and Methods

### Patient Samples

20 lung adenocarcinoma (LUAD) and 11 squamous cell carcinoma (LUSC) paraffin-embedded tumor samples from patients diagnosed between 2013 and 2016 were obtained from the Department of Pathology at Thomas Jefferson University.

### Immunohistochemistry

Immunohistochemistry staining was performed on human lung cancer samples and mouse xenografts as previously described (51). Briefly, antigen retrieval was performed in 10 mM citrate buffer, pH 6.0, for 10 minutes using a pressure cooker. Sections were blocked with 3% hydrogen peroxide for 15 minutes and blocked for endogenous biotin (Avidin-biotin kit, Biocare Medical AB972 L). Then, sections were incubated with 10% goat serum followed by primary antibody incubation. Human samples were blocked for 30 minutes at room temperature and incubated with the primary antibody overnight at 4°C; mouse xenografts were blocked overnight at 4°C and incubated with the primary antibody for 1 hour at room temperature. The primary antibodies used were anti-MCT4 (19-mer peptide sequence CSPDQKDTEGGPKEEESPV-cooh affinity purified rabbit antibody, YenZym, YZ4718; H-90, Santa Cruz sc-50329), anti-mCherry (Aviva Systems Biology, OAAF04756), anti-MCT1 (19-mer peptide sequence CSPDQKDTEGGPKEEESPV-cooh affinity purified rabbit antibody; YenZym), anti-TOMM20 (F-10, Santa Cruz sc-17764), anti-IDH3α (A-10, Santa Cruz, sc-398021), anti-GLUT1 (Millipore, 07-1401), and anti-PCK2 (or PEPCK-M, Abcam, ab70359). The biotinylated secondary antibodies used were goat anti-rabbit (Vector Laboratories, BA-1000) and goat anti-mouse IgGs (Vector Laboratories, BA-9200), followed by incubation with an avidin-horseradish peroxidase (HRP) complex (Vecstain Elite ABC kit, Vector PK-6100). Antibody reactivity was detected using liquid DAB substrate chromagen (Dako K346711-2). For staining with mouse-TOMM20 antibody on mouse tissue, the Vector M.O.M. Basic kit (Vector Laboratories, BMK-2202) was used. Counterstaining of nuclei was performed with Tacha’s hematoxylin (Biocare Medical, NM-HEM M) staining. Images were taken with an Olympus DP22 camera system attached to the microscope.

### Cell Culture

The human NSCLC cell lines A549, HCC827, NCI-H226, NCI-H520, NCI-H23, and NCI-H1299, and the human HNSCC cell lines SCC9 and SCC25 were purchased from ATCC. Regarding the genetic background of these cell lines, A549 and H23 have G12S and G12C KRAS mutations, respectively. HCC827 has an E746-A750 deletion in EGFR and a V218 deletion in TP53. H520 has a G438A mutation in TP53. H226 has no reported genetic alterations in RAS, TP53 or EGFR. H1299 is TP53 null and has a Q16K mutation in NRAS. SCC9 and SCC25 have a V274-G285 deletion and R209Kfs*6 mutation, respectively, in TP53. Human skin fibroblasts immortalized with human telomerase reverse transcriptase catalytic domain (BJ1) were purchased from Clontech, and clones were generated with green fluorescent protein (GFP) and red fluorescent protein (RFP). The C56BL/6 mouse head and neck squamous carcinoma cell lines MOC1 and MOC2 were a kind gift from Dr. Ravindra Uppaluri. A549 cells were cultured in F-12K medium (ATCC 30-2004). HCC827, H226, H520 and H23 cells were cultured in RPMI-1640 (ATCC 30-2001). SCC9 and SCC25 were cultured in DMEM:F12-K (1:1) (ATCC 30-2006) with 400 ng/mL of hydrocortisone (Sigma, H0888). NCI-H1299 and BJ1 were cultured in DMEM (Gibco 10566016). MOC1 and MOC2 were cultured in IMDM:F-12K (2:1) with 5 µg/mL of epidermal growth factor (Gibco, PHG0311), 400 ng/mL of hydrocortisone, 5 µg/mL of insulin (Sigma, I0516), and 1% Amphotericin B (Gibco, 15290018). All cells were kept in a 37°C and 5% CO_2_ humidified incubator. Culture mediums contained 100 units/ml penicillin, and 100 units/ml streptomycin (PenStrep, Corning, 30-002-CI) and 10% heat-inactivated fetal bovine serum (HI-FBS, Gibco, 161407-071), except for MOC1 and MOC2 cell lines containing 5% HI-FBS.

### Co-Culture System

Fibroblasts and carcinoma cells were co-cultured in 12-well plates or 10 cm dishes, as specified. A549 were co-cultured with BJ1 or MEFs at a 5:1 fibroblast to carcinoma cell ratio. HCC827, H226, H520, H23, SCC9 and SCC25 were co-cultured with BJ1 at a 3:1 fibroblast to carcinoma cell ratio. The total number of cells seeded per well was 1 x 10^5^, and per dish was 12 x 10^5^. As controls, monocultures of BJ1 and carcinoma cells were plated with the same total number of cells. Cells were seeded in DMEM with 10% HI-FBS and PenStrep. The following day, culture media was changed to DMEM with 10% Nu-serum (a low protein alternative to FBS; BD Biosciences, #355100) containing PenStrep. Cells were maintained in this media for 3 days.

### Mouse Embryonic Fibroblast Isolation

MEFs were isolated from genetically engineered C57BL/6 mice lacking MCT4 (MCT4-knock out) and their wild-type control (WT) (kind gift from Dr. Nancy Philp), as previously described (51). Briefly, pregnant female mice were euthanized at E14.5 by CO_2_ inhalation. Under sterile conditions, embryos were extracted, separated from the placenta and placed into a new dish with PBS for individual manipulation. Head, arms, and legs were cut off, and internal red tissue (heart and liver) was removed. The remaining embryo was minced and transferred into a tube containing 0.25% trypsin-EDTA. Cell suspension was incubated for 10 minutes at 37°C, and then MEF culture media (DMEM 10% FBS) was added to the tube. Cell suspension was let undisturbed for 5 minutes to allow large fragments to precipitate and supernatant was transferred to 10 cm Petri dishes. Media was refreshed once MEFs were seen attached to the plate. MEFs were maintained in DMEM 10% FBS Pen Strep for no longer than ten passages.

### MCT4 Downregulation in BJ1

Sg RNA lentiviral expression clones targeting SLC16A3 (HCP253996-LvSG02), Sg RNA lentiviral control for pCRISPR-LvSG02 (CCPCTR01-LvSG02), and Cas9 nuclease lentiviral expression clone 1 (CP-LvC9NU-01) were purchased from GeneCopoeia. Lentiviral vectors contain mCherry as a reporter maker. Lentiviruses were prepared according to the manufacturer’s protocols. Virus-containing media were centrifuged, filtered (0.45 µM polyethersulfone (PES) low protein filter) and stored in 1 mL aliquots at −80°C. BJ1-fibroblasts (120,000 cells/well) were plated in 12-well dishes in growth media. After 24 hours, the media was removed and replaced with 250 µl DMEM with 5% FBS, 150 µl of virus-containing media and 5 µg/ml polybrene. 24 hours post-transduction, media containing virus was removed and replaced with DMEM with 10% FCS. After infection, fibroblasts stably downregulated for MCT4 were selected with puromycin for 5 days.

### Cell Treatments

Co-cultures and single cultures were incubated with Nu-serum DMEM containing 10 mM of N-acetylcysteine (NAC) (Sigma, A7250), 5 µM BAY 87-2243 (Xcessbio, M60127) or no treatment control during the last 24 or 48 hours, as specified, before cell collection for immunofluorescence and flow cytometry analyses. NAC-containing media was refreshed every 24 hours.

### Immunofluorescence

Immunofluorescence (IF) staining was performed on cells after 4 days of culture in 12-well plates, as previously described (51). Briefly, cells were fixed with methanol, or fixed with 2% paraformaldehyde followed by methanol permeabilization, and then blocked with PBS containing 1% BSA and 0.1% Tween-20. Primary antibodies used were anti-MCT4 (19-mer peptide sequence CSPDQKDTEGGPKEEESPV-cooh affinity purified rabbit antibody, YenZym, YZ4718), anti-IDH3α (A-10, Santa Cruz, sc-398021), anti-TOMM20 (F-10, Santa Cruz, sc-17764), anti-PCK2 (or PEPCK-M, Abcam, ab70359), anti-Cytokeratin K8/18 (Fitzgerald, 20R-CP004). Secondary antibodies used were fluorochrome-conjugated anti-rabbit Alexa 568 (Invitrogen, A11036), anti-mouse Alexa 568 (Invitrogen, A11031), and anti-guinea pig Alexa 488 (Invitrogen, A11073). Cells were counter-stained with DAPI (Molecular Probes, D3571) and slides were mounted with Prolong Gold anti-fade Reagent (Molecular Probes, P36934). Images were acquired with a 40x oil objective in a Nikon A1 confocal system. Staining intensity was quantified with Image J splitting the image into RGB channels. The channel with the staining of interest was used to quantify the intensity of the staining in the regions of interest. Intensity was normalized by the number of nuclei in the region of interest.

### Proliferation Assessment

After 3 days of culture in 10 cm dishes cells were incubated with 10 µM EdU (Click-iT^®^ EdU Flow Cytometry Assay kits; Invitrogen, C10636) in DMEM 10% Nu-serum for 1 hour. After that, cells were harvested and washed with 1% BSA in PBS. Following the Click-it^®^ EdU protocol, cells were fixed, permeabilized and incubated with the Pacific Blue™ picolyl azide compound to detect EdU staining. Lastly, 1 x 10^6^ cells were stained with FxCycle™ Far Red Stain (Invitrogen, F10348). In flow cytometry analysis, nascent DNA synthesis (EdU incorporation) was detected with a Pacific Blue signal detector, and ploidy assessment (FxCycle staining) was detected with an APC signal detector. GFP-tagged BJ1 or GFP-tagged A549 were detected with a FITC signal detector. Analysis was performed with the FlowJo software.

### Apoptosis and Cell Death Assessment

After 4 days of culture in 12-well plates, cell death was quantified through propidium iodide or DAPI staining, and apoptosis was quantified through Annexin-V staining assessed by flow cytometry. Culture media and cells were collected, centrifuged, and re-suspended in 250 µl of Annexin-V binding buffer containing 8 µl/ml of Annexin-V-APC conjugate (BD Biosciences, 550474) and either 1 µl/ml of propidium iodide (SeraCare, KPL 71-04-01) or 0.1 µl/ml of DAPI. Cells were incubated at room temperature for 10 minutes in the dark. Then, cells were analyzed by flow cytometry using either a PE Texas Red signal detector (to detect propidium iodide) or a DAPI signal detector, and an APC signal detector (to detect AnnV). A GFP signal detector (for BJ1-GFP cells) or RFP signal detector (for BJ1-sgCTRL/sgMCT4 mCherry) was used to distinguish colored fibroblasts from uncolored carcinoma cells in co-culture. Analysis was performed with the FlowJo software.

### Reactive Oxygen Species Determination

After 4 days in culture in 12-well plates, cells were incubated with 5 µM CellROX™ Deep Red reagent (Invitrogen, C10422) in DMEM 10% HI-FBS for 30 minutes at 37°C. CellROX is a cell-permeable dye that is non-fluorescent in a reduced state. When in contact with intracellular ROS, it gets oxidized emitting fluorescence at absorption/emission maxima of 644-665 nm. Following incubation, cells were harvested and CellROX fluorescence was detected by flow cytometry using an APC signal detector. Either a GFP signal detector (for BJ1-GFP cells) or an RFP signal detector (for BJ1-sgCTRL/sgMCT4 mCherry) was used to distinguish between colored fibroblasts and uncolored carcinoma cells in co-culture. Analysis was performed with the FlowJo software.

### H_2_O_2_ Levels Determination

After 4 days in culture in 12-well plates, cells were incubated with Pentafluorobenzenesulfonyl fluorescein (PFBF; Cayman Chemical, 10005983) in DMEM 10% Hi-FBS for 1 hour at 37°C. PFBF is a fluorescent probe that functions by a non-oxidative mechanism. PFBF fluoresces upon specific perhydrolysis by hydrogen peroxide at excitation/emission maxima of 485-530 nm. Following incubation, cells were harvested and PFBF fluorescence was detected by flow cytometry using a FITC signal detector. An RFP signal detector (for BJ1-RFP and BJ1-sgCTRL/sgMCT4 mCherry) was used to distinguish between colored fibroblasts and uncolored carcinoma cells in co-culture. Analysis was performed with the FlowJo software.

### Animal Studies

The Institutional Animal Care and Use Committee (IACUC) approved all animal protocols, and experiments were performed following the National Institutes of Health guidelines. Male nude mice (NCI Athymic NCr-nu/nu, Charles River) and WT and MCT4-KO C57BL/6 mice (obtained from Dr. Nancy Philp’s lab) were maintained in a pathogen-free environment/barrier facility at the Sidney Kimmel Cancer Center at Thomas Jefferson University. Mouse xenografts were generated by co-injecting human carcinoma cells and fibroblasts resuspended in 100 µl of sterile PBS bilaterally into the flanks of male nude mice. Cell numbers injected were: 2 x 10^6^ HCC827 and 2 x 10^6^ BJ1-sgCTRL or BJ1-sgMCT4; 10^6^ HCC827 and 10^6^ WT or MCT4-KO MEF; 10^6^ H520 or SCC25 and 10^6^ BJ1-sgCTRL or BJ1-sgMCT4. Syngeneic tumors were generated by co-injecting mouse carcinoma cells and WT or MCT4-KO MEFs resuspended in 100 µl of sterile PBS bilaterally into the flanks of WT and MCT4-KO C57BL/6 female mice, respectively. Cell numbers injected were: 7 x 10^5^ MOC1 and 10^6^ WT or MCT4-KO MEF; 10^5^ MOC2 and 2 x 10^5^ WT or MCT4-KO MEF. Tumors were collected at different time points depending on the model (specified in the figure legends) and tumor size and weight were determined. For all experiments, tumor volume (mm^3^) was estimated using the formula V = (X^2^Y)/2, where V is the volume of the tumor, X is the length of the short axis, and Y is the length of the long axis.

### IHC Quantification

For MCT4, MCT1 and GLUT1 in mouse xenografts, the ImmunoMembrane computer-assisted pathology program was used to assess strength of membranous staining in carcinoma cells. ImmunoMembrane is a previously described and publicly available algorithm to detect and quantify cell surface markers stained by IHC; it was originally described for quantification of surface staining in breast cancer (53). Light photomicrographs were taken of representative areas from each tumor and uploaded into ImmunoMembrane at 20x or 40x magnification. ImmunoMembrane processes photomicrographs and distinguishes between “strong and complete” staining, labeled in red, or “weak and incomplete” staining, labeled in green, depending on the intensity and pattern of membranous staining. ImageJ was used to quantify the area labeled in red by ImmunoMembrane corresponding to the strong and complete staining. In ImageJ, images were split into RGB channels. The red labeling from ImmunoMembrane shows in the green channel on ImageJ. The “threshold” tool was used to select the areas of red labeling. The threshold value was maintained across all images for comparison. The “measure” tool was used to calculate the area of red labeling in pixels from the full image. Only images with high cancer/stromal ratios (>70% cancer area) were used for analysis. Quantitative analysis of TOMM20 immunohistochemistry in mouse xenografts was performed using Aperio software (Leica Biosystems, Buffalo Grove, IL). Briefly, tissue sections were scanned on a ScanScopeXT under 320 magnification with an average scan time of 120 seconds (compression quality 70). Images were analyzed using the Color Deconvolution and Colocalization Aperio Image Analysis tool. Areas of staining were color separated from hematoxylin counter-stained areas and the intensity of the staining was measured on a continuous scale. Five representative areas of tumor cells were selected for each sample (4 tumors per group) and the percent strong (3+) staining was quantified.

### Statistical Analyses

Statistical significance was examined using two-sample equal variance (homoscedastic) two-tailed Student’s t-test. Differences were considered statistically significant at p<0.05.

## Results

### Human NSCLC Expresses Markers of Metabolic Compartmentalization

**Figure 1:**
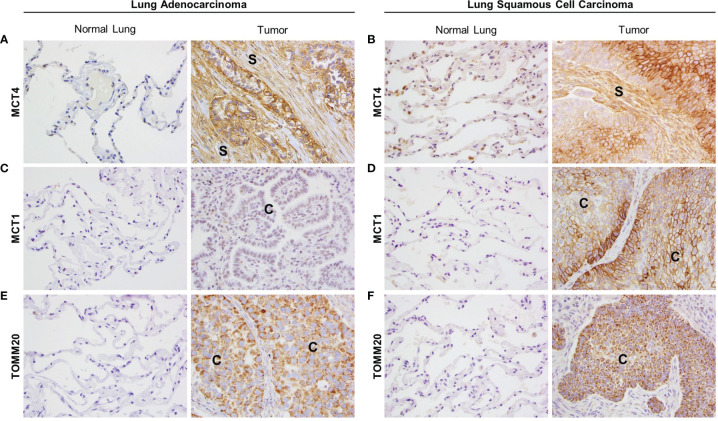
Expression of markers of metabolic compartmentalization in non-small cell lung cancer (NSCLC). Lung adenocarcinoma (LUAD) and lung squamous cell carcinoma (LUSC) patient samples were stained for MCT4, MCT1 and TOMM20 by immunohistochemistry. For each marker, representative images were taken from tumor tissue and adjacent normal lung tissue within the same sample. **(A, B)** MCT4 expression in the tumor stroma of LUAD **(A)** and LUSC **(B)** and their corresponding adjacent normal lung. **(C, D)** MCT1 expression in carcinoma cells in LUAD **(C)** and LUSC **(D)** and their corresponding adjacent normal lung. **(E, F)** TOMM20 expression in carcinoma cells in LUAD **(E)** and LUSC **(F)** and their corresponding adjacent normal lung. Images were taken at 20X. (S, stroma; C, carcinoma cells).

Primary tumor tissue samples from 20 LUAD and 11 LUSC patients were stained by IHC for several metabolic markers. Areas of adjacent normal lung tissue within the same sample were used for comparison. IHC staining in LUAD samples was visually scored by three independent researchers, including one trained pathologist, and staining in LUSC samples was quantified by a trained pathologist using the Aperio digital pathology software. Representative images for each staining are presented in **Figure 1** and the scoring results are shown in **Supplementary Table 1** (for LUAD) and **Supplementary Figure 1** (for LUSC). MCT4 showed a membranous staining pattern and was found expressed in the CAF-rich stroma in both LUAD (**Figure 1A** and **Supplementary Table 1**) and LUSC (**Figure 1B** and **Supplementary Figure 1A**), whereas no MCT4 staining was detected in the fibroblasts from the adjacent normal lung tissue (**Figures 1A, B**, **Supplementary Table 1**, and **Supplementary Figure 1A**). MCT1 also showed a membranous staining pattern and was found expressed in carcinoma cells in LUSC (**Figure 1D** and **Supplementary Figure 1B**) but not in LUAD (**Figure 1C** and **Supplementary Table 1**). Also, MCT1 expression was absent in normal lung tissue (**Figures 1C, D**, **Supplementary Table 1**, and **Supplementary Figure 1B**). TOMM20 showed a mitochondrial staining pattern, and it was found expressed in carcinoma cells both in LUAD (**Figure 1E** and **Supplementary Table 1**) and in LUSC (**Figure 1F** and **Supplementary Figure 1C**), whereas absent in normal lung tissue (**Figures 1E, F**, **Supplementary Table 1** and **Supplementary Figure 1C**). Finally, we analyzed the prognostic value of MCT4, MCT1 and TOMM20 expression in LUAD, LUSC and HNSCC from TGCA datasets (**Supplementary Figures 1D–L**). In summary, NSCLC presents markers of metabolic compartmentalization with upregulated MCT4 in the stromal compartment and upregulated MCT1 and TOMM20 in carcinoma cells. These metabolic markers are also found expressed following the same pattern in HNSCC (19), showing that at least a subset of ADT cancers have metabolic compartmentalization.

### Co-Culture Systems Reproduce Human Metabolic Compartmentalization

**Figure 2:**
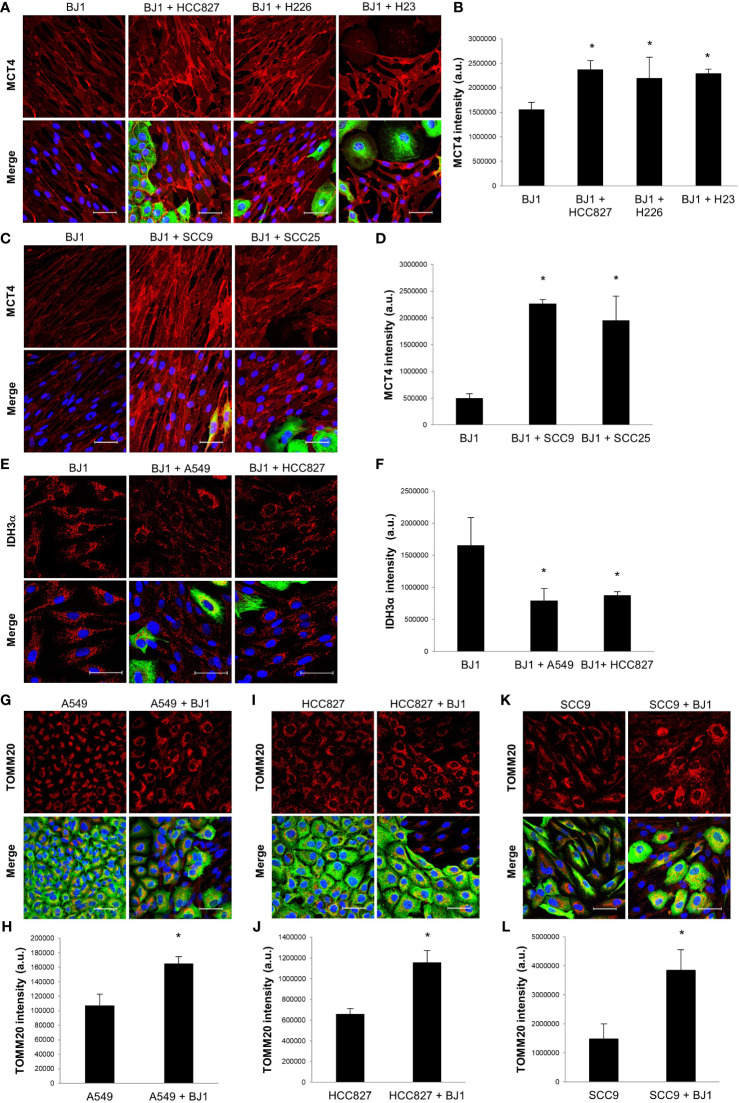
Expression of markers of metabolic compartmentalization in co-cultures of ADT carcinoma cells and fibroblasts. Human ADT carcinoma cell lines A549, HCC827, H226, H23, SCC9 and SCC25 were co-cultured with BJ1 fibroblasts for 4 days. Monocultures of carcinoma cells and BJ1 fibroblasts were maintained in parallel as controls. **(A–D)** Confocal imaging of MCT4 in monocultures of BJ1 and in co-cultures of BJ1 with the NSCLC cells HCC827, H226 and H23 **(A)** and HNSCC cells SCC9 and SCC25 **(C)**, and their respective quantification of MCT4 staining in BJ1 **(B, D)**. **(E, F)**. Confocal imaging of IDH3α in monocultures of BJ1 and in co-cultures of BJ1 with A549 and HCC827 **(E)**, and quantification of IDH3α staining in BJ1 **(F)**. **(G–L)** Confocal imaging of TOMM20 in monocultures and co-cultures of A549 **(G)**, HCC827 **(I)**, and SCC9 **(K)** with BJ1, and quantification of TOMM20 staining in A549 **(H)**, HCC827 **(J)** and SCC9 **(L)**. Confocal microscopy images were acquired at the 40X magnification, with 1.5x zoom in panel **(E)**. For all images, MCT4, IDH3α and TOMM20 staining are shown in red, carcinoma cells are shown in green (K8/18 staining), and nuclei are shown in blue (DAPI). For all markers, red staining was quantified using ImageJ. Student’s t-test was used for statistical analyses (*p<0.05). (a.u., arbitrary units. Scale bar = 50 μm).

In order to reproduce metabolic compartmentalization in experimental models *in vitro*, we co-cultured human NSCLC and HNSCC cell lines with the human fibroblast cell line BJ1. Immunofluorescent staining and confocal imaging were performed to determine whether co-culturing conditions could upregulate the expression of markers of metabolic compartmentalization in carcinoma cells and fibroblasts, in comparison to their respective monocultures. Co-cultures of BJ1 fibroblasts with the NSCLC carcinoma cell lines HCC827, H226, H23 and H1299 significantly upregulated the expression of MCT4 in BJ1, compared to BJ1 in monoculture (**Figures 2A, B**; **Supplementary Figures 2A, B**). Co-cultures of BJ1 with the NSCLC carcinoma cell lines A549 and H520 showed a trend towards MCT4 upregulation in BJ1, however the differences were not statistically significant (**Supplementary Figures 2C, D**). Similarly to NSCLC, BJ1 co-cultured with the HNSCC carcinoma cell lines SCC9 and SCC25 also upregulated MCT4 expression, compared to BJ1 in monoculture (**Figures 2C, D**). MCT4 expression in all carcinoma cell lines either in monoculture or co-culture was heterogeneous and no significant changes in MCT4 levels were observed (data not shown). In NSCLC, BJ1 co-cultured with A549 and HCC827 downregulated the expression of IDH3α, another marker of glycolysis in CAFs, compared to BJ1 in monoculture (**Figures 2E, F**), whereas no significant changes in IDH3α in carcinoma cells were observed (data not shown). We also assessed the expression of IDH3α in the stroma of human NSCLC patient samples. IHC staining revealed that IDH3α was mainly expressed in lung carcinoma cells, whereas it was absent in the CAF-rich stroma in NSCLC (**Supplementary Figure 2E**). Additionally, transwell co-cultures of BJ1 with carcinoma cells, where cells are not in direct contact, and cultures of BJ1 with conditioned media from NSCLC or HNSCC co-cultures were performed to study markers of glycolytic metabolism in BJ1 by western blot. BJ1 increased MCT4 expression in transwell co-culture with A549, H520 and SCC25 (**Supplementary Figures 2F–H**), and decreased IDH3α expression in transwell co-culture with SCC9 and SCC25 (**Supplementary Figure 2I**), all compared to BJ1 in monoculture. Moreover, culturing BJ1 with conditioned medias from various co-cultures was sufficient to induce downregulation of IDH3α, upregulation of MCT4, and upregulation of LDH-V, another HIF1α-driven glycolytic enzyme, compared to untreated BJ1 (**Supplementary Figure 2J**). Co-culture of NSCLC carcinoma cell lines A549 and HCC827 with BJ1 increased the expression of TOMM20 in both carcinoma cells lines (**Figures 2G–J**), compared to their respective monocultures. Conversely, TOMM20 expression was decreased in BJ1 in co-culture with both carcinoma cell lines compared to BJ1 in monoculture (data not shown). The HNSCC cell line SCC9 also significantly upregulated TOMM20 expression in the presence of BJ1, compared to its monoculture (**Figures 2K, L**), and SCC25 showed a trend towards increased TOMM20 expression in co-culture, however it did not reach statistical significance (**Supplementary Figures 2K, L**). Transwell co-culture of A549 and SCC25 with BJ1 also increased TOMM20 expression in both carcinoma cell lines, compared to their monocultures (**Supplementary Figures 2M, N**). Altogether, our data shows that co-culture models of ADT carcinoma cells with fibroblasts upregulate markers of metabolic compartmentalization as seen in human tumors, proving evidence of their validity to study the metabolism of these diseases *in vitro*.

### PEPCK-M Is Upregulated in Glycolytic Fibroblasts

**Figure 3:**
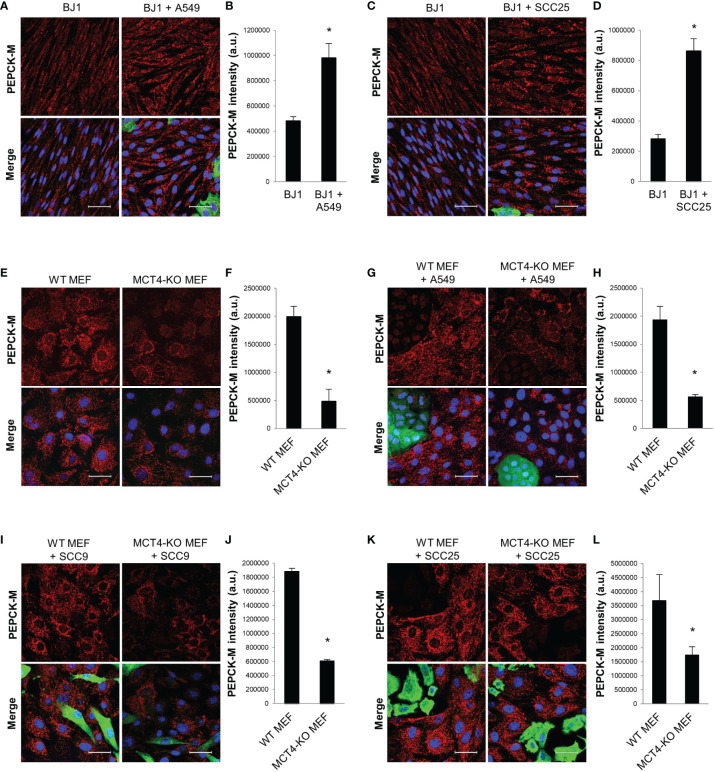
PEPCK-M expression in glycolytic fibroblasts. BJ1 and WT or MCT4-KO MEF were co-cultured with ADT carcinoma cells. Monocultures of BJ1 and MEF were maintained in parallel as controls. **(A–D)** Confocal imaging of PEPCK-M in monocultures and in co-cultures of BJ1 with A549 **(A)** and SCC25 **(C)**, and their respective quantification of PEPCK-M staining in BJ1 **(B, D)**. **(E, F)** Confocal imaging of PEPCK-M in monocultures of WT and MCT4-KO MEF **(E)** and quantification of PEPCK-M staining **(F)**. **(G–L)** Confocal imaging of PEPCK-M in WT and MCT4-KO MEF in co-culture with A549 **(G)**, SCC9 **(I)** and SCC25 **(K)**, and their respective quantification of PEPCK-M staining in MEF **(H, J, L)**. Confocal microscopy images were acquired at the 40X magnification. For all images, PEPCK-M staining is shown in red, carcinoma cells are shown in green (K8/18 staining), and nuclei are shown in blue (DAPI). PEPCK-M staining was quantified using ImageJ. Student’s t-test was used for statistical analyses (*p<0.05). (a.u., arbitrary units. Scale bar = 50 μm).

PEPCK-M is a master regulator of central carbon metabolism (34, 36–38). We aimed to determine whether PEPCK-M could be involved in the glycolytic reprogramming of fibroblasts in response to carcinoma cells. BJ1 fibroblasts showed upregulation of PEPCK-M when co-cultured with A549 (**Figures 3A, B**; **Supplementary Figure 3A**) and SCC25 (**Figures 3C, D**; **Supplementary Figure 3B**) carcinoma cells, compared to BJ1 in monoculture. Moreover, conditioned media from various co-cultures was also capable to increase the expression of PEPCK-M in BJ1 fibroblasts (**Supplementary Figure 3C**). MCT4 is a rate-limiting step of glycolysis, and its downregulation causes a reduction in glycolytic flux (30). To determine whether PEPCK-M expression was modulated by glycolysis in fibroblasts, we assessed PEPCK-M expression in WT and MCT4-KO MEFs. There was decreased expression of PEPCK-M in MCT4-KO MEFs compared to WT MEF in monoculture (**Figures 3E, F**; **Supplementary Figure 3D**). Moreover, MCT4-KO MEF co-cultured with A549 (**Figures 3G, H**), SCC9 (**Figures 3I, J**) and SCC25 (**Figures 3K, L**) carcinoma cells were unable to upregulate PEPCK-M expression to similar levels as those observed in co-cultured WT MEFs. Additionally, we saw that PEPCK-M was upregulated in BJ1 fibroblasts exposed to cigarette smoke (**Supplementary Figure 3E**), a condition that has been previously described to induce glycolysis and MCT4 in fibroblasts (51). We also assessed the expression of PEPCK-M in NSCLC patient samples and observed that PEPCK-M is highly expressed both in lung carcinoma cells and in stromal cells in NSCLC (**Supplementary Figure 3F**). Altogether, our data shows that PEPCK-M is upregulated in glycolytic fibroblasts in co-culture and in human NSCLC, and that its expression is modulated by MCT4.

### Co-Culture Conditions Increase Features of Aggressiveness in Carcinoma Cells

**Figure 4:**
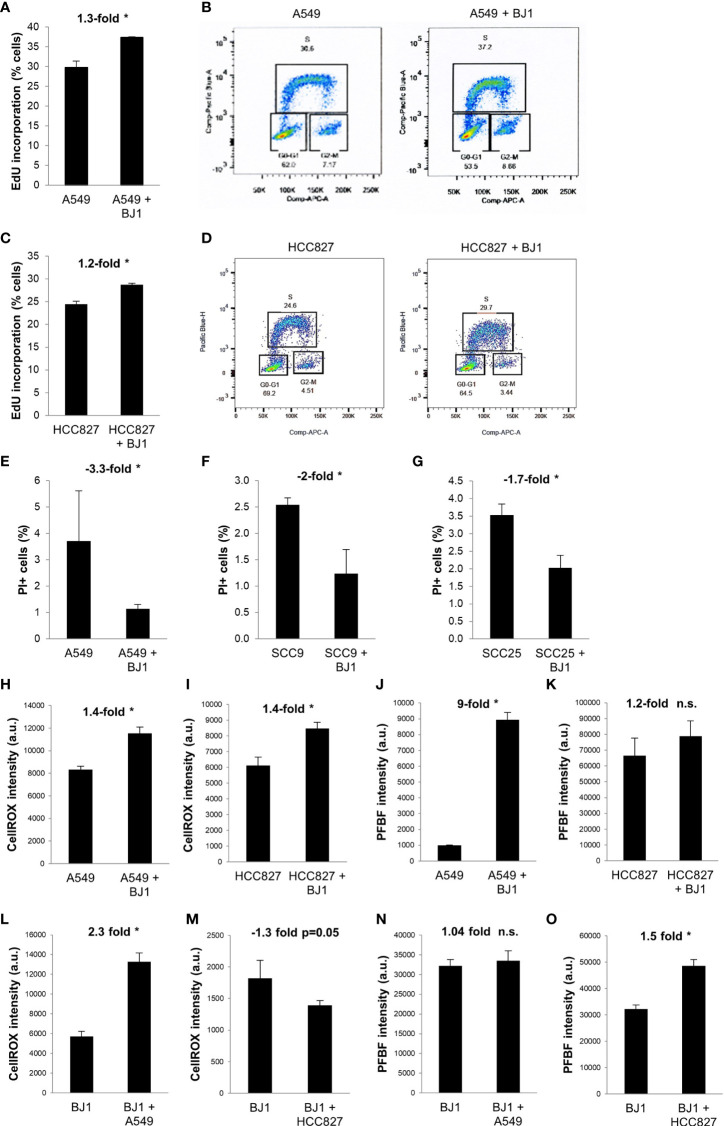
Effects of fibroblasts on ADT carcinoma cell aggressiveness. Human ADT carcinoma cells were monocultured or co-cultured with GFP-tagged BJ1 and proliferation, cell death and oxidative stress levels were assessed by flow cytometry. **(A, C)** Percentage of A549 **(A)** and HCC827 **(C)** in the DNA synthesis phase, measured by 5-ethynyl-2’-deoxyuridine (EdU) incorporation. **(B, D)** Representative flow cytometry plots showing percentage of A549 **(B)** and HCC827 **(D)** in each phase of the cell cycle, and gating strategy for EdU (Pacific Blue) and FxCycle (APC) staining. **(E–G)** Percentage of cell death as measured by propidium iodide (PI) staining in A549 **(E)**, SCC9 **(F)** and SCC25 **(G)**. **(H, I)** Reactive oxygen species (ROS) levels, as measured by the CellROX probe, in A549 **(H)** and HCC827 **(I)**. **(J, K)** Hydrogen peroxide (H_2_O_2_) levels, as measured by the PFBF probe, in A549 **(J)** and HCC827 **(K)**. **(L, M)** ROS levels in BJ1 in monoculture or in co-culture with A549 **(L)** and HCC827 **(M)**. **(N, O)** H_2_O_2_ levels in BJ1 in monoculture or in co-culture with A549 **(N)** and HCC827 **(O)**. Student’s t-test was used for statistical analyses (* p<0.05). (a.u., arbitrary units). ns, not significant.

We next sought to determine whether co-cultures of carcinoma cells and fibroblasts, conditions that alter metabolism in both cell compartments, could promote carcinoma cell aggressiveness. Increased proliferation, decreased cell death, oxidative stress, enhanced migratory capabilities, and stemness are some hallmarks of carcinoma cell aggressiveness. Flow cytometry assessment of EdU incorporation and DNA ploidy revealed that NSCLC carcinoma cells had increased proliferation rates in the presence of fibroblasts. Co-culture conditions increased the percentage of A549 (**Figures 4A, B**) and HCC827 (**Figures 4C, D**) cells incorporating EdU, compared to monocultures of carcinoma cells, indicating an increase in the number of carcinoma cells undergoing nascent DNA synthesis (S phase). Flow cytometry assessment of PI staining showed that co-culture conditions led to decreased carcinoma cell death in A549 (**Figure 4E**), SCC9 (**Figure 4F**) and SCC25 (**Figure 4G**) cells, compared to monocultures of carcinoma cells. Oxidative stress levels in co-cultures were assessed by flow cytometric analysis of the CellROX reporter and the PFBF probe. The CellROX reporter quantifies unspecific intracellular ROS, and the PFBF probe specifically measures the levels of hydrogen peroxide (H_2_O_2_), the principal ROS involved in signaling due to its high permeability across cell membranes and ability to diffuse. Co-culture conditions increased the levels of ROS in both A549 (**Figure 4H**) and HCC827 (**Figure 4I**) cells. However, only A549 cells (**Figure 4J**), but not HCC827 (**Figure 4K**), had significantly augmented levels of H_2_O_2_ upon co-culture. In fibroblasts, ROS levels were increased in BJ1 co-cultured with A549 (**Figure 4L**), but not with HCC827 (**Figure 4M**). Inversely, H_2_O_2_ levels did not increase in BJ1 co-cultured with A549 cells (**Figure 4N**), but they did so in BJ1 co-cultured with HCC827 (**Figure 4O**). The migratory abilities of carcinoma cells were assessed with a transwell assay. A549 (**Supplementary Figures 4A, B**), but not HCC827 (**Supplementary Figure 4C**) nor SCC25 (**Supplementary Figure 4D**), showed increased migration through the transwell membrane pores in response to BJ1, compared to normal growth media. Markers of stemness in carcinoma cells in co-cultures versus monocultures were also assessed, however no differences were seen (data not shown). Altogether, our results demonstrate that co-culture conditions increase some features of carcinoma cell aggressiveness with increased proliferation and oxidative stress and decreased cell death.

### BAY 87-2243 Partially Rescues the CAF Phenotype and Induces Carcinoma Cell Apoptosis

**Figure 5:**
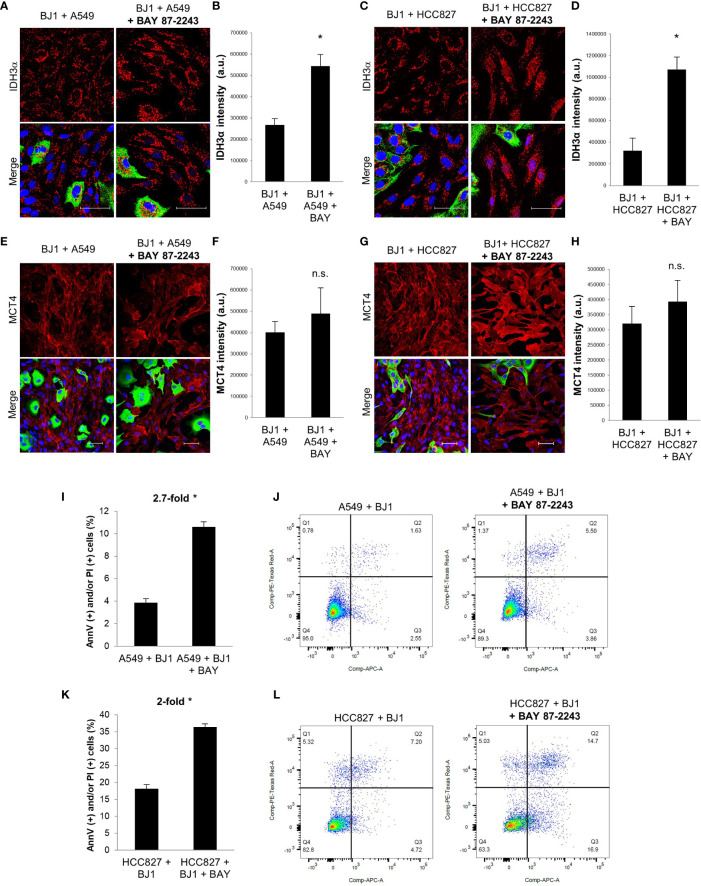
Effects of HIF-1α inhibition on fibroblast metabolism and carcinoma cell aggressiveness. Co-cultures of ADT carcinoma cells and BJ1 were treated with the HIF-1α inhibitor BAY 87-2243 or vehicle control for 48 hours. **(A–D)** Confocal imaging of IDH3α in co-cultures of BJ1 with A549 **(A)** and HCC827 **(C)** treated or untreated with BAY 87-2243, and their respective quantification of IDH3α staining in BJ1 **(B, D)**. **(E–H)** Confocal imaging of MCT4 in co-cultures of BJ1 with A549 **(E)** and HCC827 **(G)** treated or untreated with BAY 87-2243, and their respective quantification of MCT4 staining in BJ1 fibroblasts **(F, H)**. Confocal microscopy images were acquired at the 40X magnification, with 1.5x zoom in panels **(A, C)**. For all images, IDH3α and MCT4 staining are shown in red, carcinoma cells are shown in green (K8/18 staining), and nuclei are shown in blue (DAPI). IDH3α and MCT4 staining were quantified using ImageJ. **(I–L)** Flow cytometry assessment of percentage of carcinoma cells undergoing apoptosis (AnnV staining) and cell death (PI staining) in co-cultured A549 **(I, J)** and HCC827 **(K, L)** untreated or treated with BAY 87-2243 with representative flow cytometry plots showing gating strategy for AnnV (APC) and PI (PE) staining. Student’s t-test was used for statistical analyses (*p<0.05). (a.u., arbitrary units. Scale bar = 50 μm).

Given that HIF-1α is a main driver of the glycolytic and catabolic state with secretion of monocarboxylates in CAFs (9), we next aimed to assess whether HIF-1α inhibition with the drug BAY 87-2243 could have an impact on the metabolism of fibroblasts and carcinoma cell aggressiveness in ADT co-cultures. We treated co-cultures of A549 or HCC827 and BJ1 fibroblasts with BAY 87-2243 and assessed IDH3α and MCT4 expression by confocal imaging. BAY 87-2243 increased IDH3α expression in BJ1 fibroblasts in co-culture with A549 (**Figures 5A, B**) and HCC827 (**Figures 5C, D**), compared to untreated co-cultures. However, BAY 87-2243 was not able to downregulate MCT4 expression in BJ1 in co-culture (**Figures 5E–H**). Treatment with BAY 87-2243 resulted in increased carcinoma cell apoptosis and death in A549 (**Figures 5I, J**) and HCC827 (**Figures 5K, L**) in co-culture with BJ1, compared to untreated co-cultures. In summary, our data demonstrate that targeting HIF-1α with BAY 87-2243 has effects on both fibroblasts and carcinoma cells in co-culture. In fibroblasts it upregulates IDH3α expression, partially reverting the CAF phenotype, and in carcinoma cells it decreases apoptosis and cell death, a hallmark of carcinoma cell aggressiveness.

### Downregulation of Fibroblast MCT4 Reduces Features of Carcinoma Cell Aggressiveness

**Figure 6:**
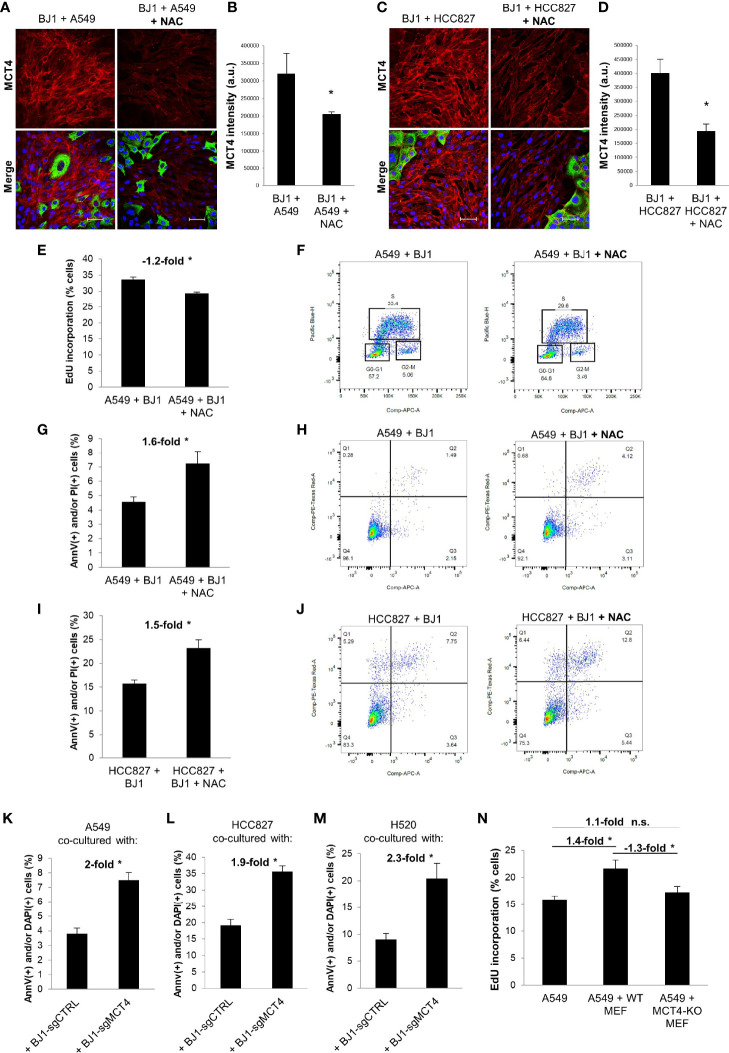
Effects of ROS neutralization and MCT4 downregulation on fibroblast metabolism and ADT carcinoma cell aggressiveness. Co-cultures of ADT carcinoma cells with BJ1 were treated with N-acetyl cysteine (NAC) for 24 hours for proliferation assessments, and for 48 hours for confocal imaging and apoptosis assessment. **(A–D)** Confocal imaging of MCT4 in co-cultures of BJ1 with A549 **(A)** and HCC827 **(C)** treated or untreated with NAC, and respective quantification of MCT4 staining in BJ1 **(B, D)**. Confocal microscopy images were acquired at the 40X magnification. For all images, MCT4 staining is shown in red, carcinoma cells are shown in green (K8/18 staining), and nuclei are shown in blue (DAPI). MCT4 staining was quantified using ImageJ. **(E, F)** Flow cytometry quantification of percentage of A549 cells in the DNA synthesis phase, measured by 5-ethynyl-2’-deoxyuridine (EdU) incorporation **(E)**, and gating strategy for EdU (Pacific Blue) and FxCycle (APC) staining **(F)**. **(G–J)** Flow cytometry assessment of percentage of carcinoma cells undergoing apoptosis (AnnV staining) and cell death (PI staining) in co-cultured A549 **(G, H)** and HCC827 **(I, J)** cells untreated or treated with NAC with representative flow cytometry plots showing gating strategy for AnnV (APC) and PI (PE) staining. **(K–M)** ADT carcinoma cells were co-cultured with mCherry-tagged BJ1 with downregulated MCT4 expression (BJ1-sgMCT4) or their control counterparts (BJ1-sgCTRL) and apoptosis (AnnV staining) and cell death (DAPI staining) was assessed in A549 **(K)**, HCC827 **(L)** and H520 **(M)** by flow cytometry. **(N)** GFP-tagged A549 were monocultured or co-cultured with WT or MCT4-KO MEFs and proliferation rates in A549 were assessed by flow cytometric analysis of EdU incorporation. Student’s t-test was used for statistical analyses (*p<0.05). (a.u., arbitrary units. Scale bar = 50 μm). ns, not significant.

As we observed that co-culture conditions increase oxidative stress, which is an inducer of glycolysis and MCT4 in CAFs (9, 54), we next aimed to target ROS and assess its effects on fibroblast metabolism and carcinoma cell aggressiveness in ADT co-cultures. N-acetylcysteine (NAC) is a potent antioxidant that has been shown to have anti-cancer effects in numerous preclinical models (55–58). Therefore, we treated co-cultures of A549 or HCC827 and BJ1 fibroblasts with NAC to assess its effects on oxidative stress, MCT4 expression and carcinoma cell proliferation and apoptosis. NAC treatment decreased levels of H_2_O_2_ in A549 (**Supplementary Figure 5A**) and HCC827 (**Supplementary Figure 5B**) carcinoma cells and in BJ1 fibroblasts in co-culture (**Supplementary Figures 5C, D**), compared to untreated controls. Cisplatin treatment was used as a positive control for induction of H_2_O_2_ in these co-cultures (**Supplementary Figures 5A–D**). NAC reduced the expression of MCT4 in BJ1 fibroblasts in co-culture with A549 (**Figures 6A, B**), HCC827 (**Figures 6C, D**) and H520 (**Supplementary Figure 5E**), compared to untreated co-cultures. NAC treatment decreased EdU incorporation (or S phase) in A549 cells in co-culture (**Figures 6E, F**), but not in HCC827 cells (**Supplementary Figure 5F**), compared to untreated co-cultures. Moreover, NAC treatment induced apoptosis and cell death rates in co-cultured A549 (**Figures 6G, H**) and HCC827 (**Figures 6I, J**), compared to untreated co-cultures. These data demonstrate that disrupting the REDOX state of the co-cultures with an antioxidant has effects both in fibroblasts, by lowering MCT4, and in carcinoma cells, by reducing proliferation and inducing apoptosis and cell death.

We next aimed to determine whether MCT4 downregulation in co-cultured fibroblasts could exert effects on carcinoma cell aggressiveness. To test that, we co-cultured A549 and HCC827 cells with BJ1 fibroblasts genetically manipulated to downregulate MCT4 (BJ1-sgMCT4) and their control counterparts (BJ1-sgCTRL). Confocal imaging showed that BJ1-sgMCT4 had significant downregulation of MCT4, compared to BJ1-sgCTRL, in co-culture with A549 and HCC827 (**Supplementary Figures 5G, H**). Apoptosis and cell death assessment revealed that A549 (**Figure 6K**; **Supplementary Figure 5I**), HCC827 (**Figure 6L**; **Supplementary Figure 5J**) and H520 (**Figure 6M**; **Supplementary Figure 5K**) cells co-cultured with BJ1-sgMCT4 had increased rates of apoptosis and cell death, compared to co-cultures with BJ1-sgCTRL. Moreover, proliferation was assessed in co-cultures of A549 cells with either WT MEF or MCT4-KO MEF. WT MEF, but not MCT4-KO MEF, induced proliferation in co-cultured A549 cells, compared to A549 cells in monoculture, as seen by an increase in the percentage of A549 cells incorporating EdU (**Figure 6N**). Altogether, our data demonstrate that fibroblast MCT4 is a driver of carcinoma cell aggressiveness, and that the detrimental effect of NAC on carcinoma cells may be partially mediated by the downregulation of MCT4 in fibroblasts.

### Downregulation of Stromal MCT4 Reduces Tumor Growth in Vivo

**Figure 7:**
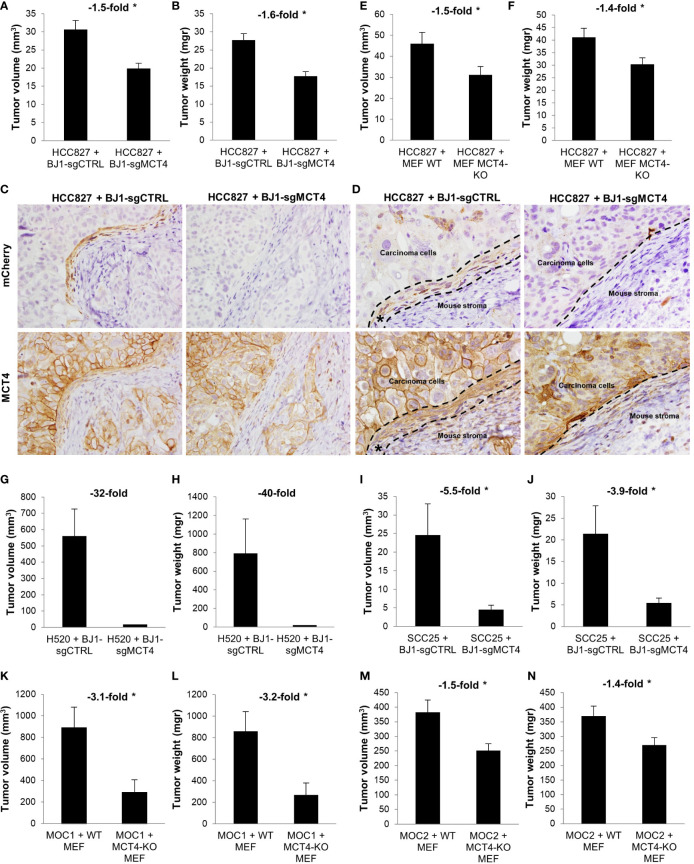
Effects of fibroblast MCT4 on tumor growth. **(A–J)** Tumor xenografts were generated by co-injecting human ADT carcinoma cells with fibroblasts expressing or lacking MCT4 into nude mice. **(A)** Tumor volume and **(B)** tumor weight of HCC827 + BJ1-sgCTRL/sgMCT4 xenografts harvested at 2.5 weeks post-implantation. **(C, D)** Immunohistochemical assessment of mCherry (to detect BJ1) and MCT4 expression in HCC827 + BJ1-sgCTRL/sgMCT4 tumors, and image acquisition at 40x **(C)** and 60x **(D)**. In panel **(D)** the area of carcinoma cells, mouse stroma, and injected mCherry-expressing BJ1 (*) are marked. **(E)** Tumor volume and **(F)** tumor weight of HCC827 + WT/MCT4-KO MEF xenografts harvested at 4 weeks post-implantation. **(G)** Tumor volume and **(H)** tumor weight of H520 + BJ1-sgCTRL/sgMCT4 xenografts harvested at 2 months post-implantation. **(I)** Tumor volume and **(J)** tumor weight of SCC25 + BJ1-sgCTRL/sgMCT4 xenografts harvested at 6 weeks post-implantation. **(K–N)** Syngeneic tumors were generated by co-injecting C57BL/6 carcinoma cells with WT or MCT4-KO MEFs into WT or MCT4-KO C57BL/6 mice. **(K)** Tumor volume and **(L)** tumor weight of MOC1 + WT/MCT4-KO MEF tumors harvested at 7 weeks post-implantation. **(M)** Tumor volume and **(N)** tumor weight of MOC2 + WT/MCT4-KO MEF tumors harvested at 3 weeks post-implantation. Student’s t-test was used for statistical analyses (*p<0.05). (a.u., arbitrary units).

We have shown that ADT human cancers have high levels of MCT4 in the CAF-rich stromal compartment (**Figures 1A, B**) (19). Also, in *in vitro* co-cultures we have demonstrated that fibroblast MCT4 is a driver of ADT carcinoma cell aggressiveness (**Figures 6K–N**). Therefore, we next aimed to investigate the effects of fibroblast MCT4 in ADT tumor growth *in vivo*. Four different mouse xenografts models were generated by co-injecting human NSCLC and HNSCC carcinoma cell lines with BJ1-sgCTRL and BJ1-sgMCT4, or with WT MEF and MCT4-KO MEF, into the flanks of nude mice. In NSCLC, co-injection of HCC827 with BJ1-sgMCT4 decreased tumor growth starting at 2 weeks post-injection, compared to co-injection with BJ1-sgCTRL (**Supplementary Figure 6A**). Tumors generated from the co-injection of HCC827 with BJ1-sgMCT4 had 50% less volume (**Figure 7A**) and had 60% less weight (**Figure 7B**) than tumors generated by co-injection with BJ1-sgCTRL. Interestingly, HCC827+BJ1-sgMCT4 tumors had reduced infiltrative stroma than HCC827+BJ1-sgCTRL tumors (**Supplementary Figures 6B, C**). Generally, co-injection of carcinoma cells with fibroblasts generates tumors with increased stromal infiltrate, compared to homotypic injection of carcinoma cells alone. Therefore, we analyzed whether there was any difference in the time fibroblasts persist in the tumors after injection between BJ1-sgCTRL and BJ1-sgMCT4 that could explain the difference in the amount of infiltrative stroma. We performed IVIS imaging on days 3 and 7 post-co-injection to detect the mCherry-labeled BJ1-sgCTRL and BJ1-sgMCT4 fibroblasts. On day 3 there were already fewer BJ1-sgMCT4 fibroblasts, compared to BJ1-sgCTRL, in the tumors (**Supplementary Figures 6D, E**). This difference was even more pronounced on day 7 where there were almost no BJ1-sgMCT4 fibroblasts present in the tumors (**Supplementary Figures 6F, G**). Additionally, we stained the tumors for mCherry and MCT4 to detect any remaining injected BJ1 fibroblasts in the tumor and assess their MCT4 expression. mCherry staining revealed the presence of the originally injected BJ1-sgCTRL in the tumors in higher numbers than BJ1-sgMCT4 (**Figure 7C**, upper panel). Both BJ1-sgCTRL and BJ1-sgMCT4 fibroblasts were found sparsely within the cancer areas, however BJ1-sgCTRL, but not BJ1-sgMCT4, were also found forming a rim of cells at the edge of carcinoma cell nests (**Figures 7C, D**, upper panel). These BJ1-sgCTRL adjacent to carcinoma cells expressed high levels of MCT4 (**Figures 7C, D**, lower panel). MCT4 was equally expressed in the infiltrated mouse stroma in both groups. HCC827 were also co-injected with either WT or MCT4-KO MEF and, similarly to the previous model, tumors generated from the co-injection of HCC827 with MCT4-KO MEF were 50% smaller (**Figure 7E**) and of 40% less weight (**Figure 7F**). In another NSCLC model, co-injection of H520 with BJ1-sgMCT4 had a poorer engraftment rate, compared to co-injection with BJ1-sgCTRL (**Supplementary Figure 6H**). Only one tumor grew in the H520+BJ1-sgMCT4 group, and it was of considerably smaller size and weight than any of the tumors grown in the H520+BJ1-sgCTRL group (**Figures 7G, H**). In HNSCC, co-injection of SCC25 with BJ1-sgMCT4 also impaired tumor growth, compared to co-injection with BJ1-sgCTRL (**Figures 7I, J**). Finally, we used two syngeneic models of HNSCC, MOC1 and MOC2, which were co-injected with either WT or MCT4-KO MEF into the flanks of WT or MCT4-KO C57BL/6 mice, respectively. In both MOC1 (**Figures 7K, L**) and MOC2 (**Figures 7M, N**) models, co-injection of MOC cells with MCT4-KO MEF into MCT4-KO mice generated smaller tumors by volume and weight than their WT counterparts. Altogether, these data demonstrate that MCT4 expression in the stroma is a driver of tumor growth in ADT cancers.

### Downregulation of Fibroblast MCT4 Changes Tumor Metabolism

**Figure 8:**
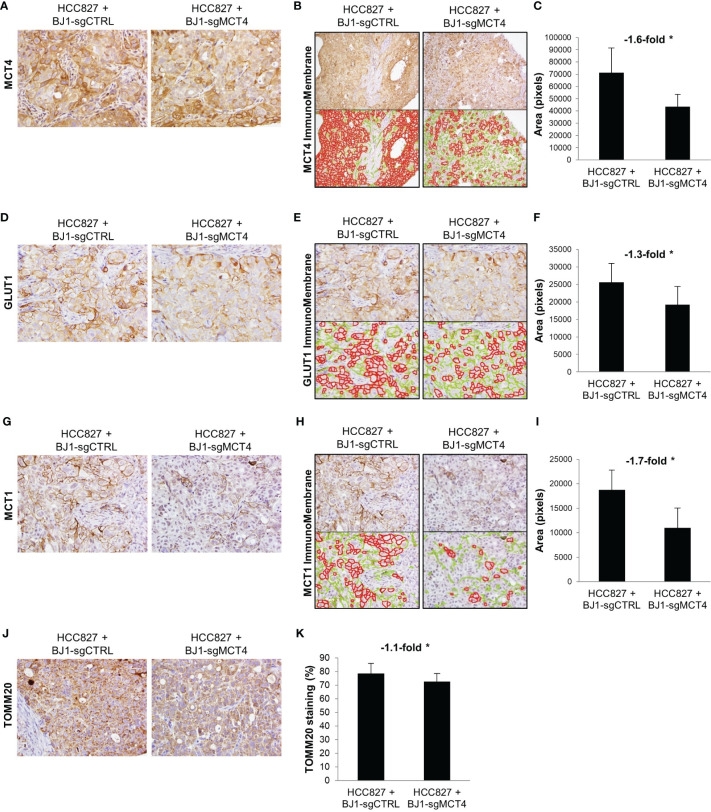
Effects of fibroblast MCT4 on tumor metabolism. The metabolic markers MCT4, GLUT1, MCT1 and TOMM20 were assessed by immunohistochemical staining in tumor xenografts generated from the co-injection of HCC827 with BJ1-sgCTRL or BJ1-sgMCT4. Representative IHC images of MCT4 **(A)**, GLUT1 **(D)**, MCT1 **(G)** and TOMM20 **(J)** staining. Membranous staining of MCT4 **(B, C)**, GLUT1 **(E, F)** and MCT1 **(H, I)** in HCC827 cells was identified with the ImmunoMembrane software and quantified with ImageJ. The ImmunoMembrane software detects and labels strong membranous staining in red and weak staining in green. Only staining labeled in red was quantified by ImageJ as the area in pixels of the total image covered by the red labeling. Mitochondrial staining of TOMM20 on HCC827 cells was quantified by Aperio **(K)**. Representative images for each group are shown. All IHC images were acquired at 40X, except for MCT4 quantification in panel **(B)** that is 20X. Student’s t-test was used for statistical analyses (*p<0.05).

Next, we determined whether expression of MCT4 in fibroblasts influences carcinoma cell metabolism *in vivo*. To assess this, we stained the xenografts generated from the co-injections of HCC827 with either BJ1-sgCTRL or BJ1-sgMCT4 for the metabolic markers MCT4, GLUT1, MCT1 and TOMM20. The membranous staining pattern of MCT4, GLUT1 and MCT1 was quantified by ImmunoMembrane and the mitochondrial staining pattern of TOMM20 was quantified by Aperio in carcinoma cell areas. Representative areas of staining and quantification are shown. MCT4 was abundantly expressed in HCC827, as seen human NSCLC, however its membrane expression in was decreased in co-injections of HCC827 with BJ1-sgMCT4, compared to BJ1-sgCTRL (**Figures 8A–C**, **Supplementary Figure 7A**). GLUT1, the main glucose importer in the plasma membrane and rate limiting step of glycolysis, was not as highly expressed as MCT4 in HCC827, however its expression was also decreased in HCC827 co-injected with BJ1-sgMCT4 (**Figures 8D–F**; **Supplementary Figure 7B**). MCT1 was found scarcely expressed in HCC827, similarly to human LUAD where MCT1 expression is low (**Figure 1C**). Co-injections of HCC827 with BJ1-sgMCT4 had lower expression of MCT1 in carcinoma cells than co-injections with BJ1-sgCTRL (**Figures 8G–I**; **Supplementary Figure 7C**). TOMM20 was highly expressed in HCC827, as seen in carcinoma cells in human NSCLC (**Figure 1E**), and its expression was decreased in HCC827 co-injected with BJ1-sgMCT4 (**Figures 8J, K**). Additionally, MCT4, MCT1 and GLUT1 expression were assessed in xenografts generated from the co-injections of HCC827 with either WT or MCT4-KO MEF. Consistently with the previous model, MCT4, MCT1 and GLUT1 were decreased in HCC827 cells co-injected with MCT4-KO MEF, compared to WT MEF (**Supplementary Figure 7D**). In conclusion, MCT4 expression in fibroblasts can modulate metabolism in carcinoma cells in co-injection models of ADT cancers.

### Inhibition of OXPHOS Decreases Carcinoma Cell Aggressiveness in Co-Culture and Co-Injection Models

We have shown that co-cultures and co-injections of ADT carcinoma cells with MCT4-expressing fibroblasts increase aggressiveness and mitochondrial metabolism in carcinoma cells and overall tumor growth. Therefore, we sought to determine whether increased OXPHOS in carcinoma cells was a mechanism by which fibroblast MCT4 drives carcinoma cell malignancy. To assess this, we used the OXPHOS inhibitors metformin and phenformin, which target the complex I of the mitochondrial respiratory chain. HCC827 cells in monoculture and in co-culture with BJ1 fibroblasts were treated with metformin. We observed that HCC827 in co-culture, which have increased mitochondrial metabolism compared to the monoculture (**Figures 2G, H**), were more sensitive to OXPHOS inhibition than HCC827 in monoculture, as seen by a reduction in the MitoTracker staining, which labels mitochondrial membrane potential (**Supplementary Figure 8A**), and an increase in apoptosis and cell death rates (**Supplementary Figure 8B**). Mice co-injected with HCC827 and BJ1-sgCTRL or BJ1-sgMCT4 were treated with oral metformin to assess whether co-injections with BJ1-sgCTRL fibroblasts, which have higher mitochondrial metabolism in the carcinoma compartment (**Figures 8G–K**), were also more sensitive to OXPHOS inhibition. Although it did not reach statistical significance, co-injections of HCC827 with BJ1-sgCTRL treated with metformin had a greater reduction in tumor growth than co-injections with BJ1-sgMCT4 (**Supplementary Figure 8C**). We performed the same experiment using phenformin, which is a more potent OXPHOS inhibitor than metformin. In this case, phenformin did significantly reduce tumor growth in HCC827+BJ1-sgCTRL co-injections, whereas it did not in HCC827+sgMCT4 co-injections (**Supplementary Figure 8D**). Consistently, phenformin treatment *in vitro* increased carcinoma cell apoptosis and cell death in HCC827 co-cultured with BJ1-sgCTRL, compared to co-culture with BJ1-sgMCT4 (**Supplementary Figure 8E**). Finally, we assessed the effects of metformin treatment on carcinoma cell metabolism in tumors *in vivo*. Prior to sacrifice, mice were injected with 2-NBDG, a fluorescent glucose analogue that cannot be metabolized through the first step of glycolysis, to measure glucose uptake in the carcinoma cell compartment. 2-NBDG was assessed in EPCAM-stained carcinoma cells by flow cytometry. HCC827+BJ1-sgCTRL tumors treated with metformin showed increased glucose uptake in HCC827 compared to untreated tumors, consistent with a compensatory increase in glycolysis due to OXPHOS inhibition by metformin (**Supplementary Figure 8F**). HCC827+BJ1-sgMCT4 tumors at baseline had increased glucose uptake in HCC827 cells compared to HCC827+BJ1-sgCTRL tumors (**Supplementary Figure 8F**), consistently with the previously observed decreased mitochondrial profile of HCC827+BJ1-sgMCT4 tumors (**Figures 8G–K**). Metformin treatment also increased glucose uptake in HCC827 co-injected with BJ1-sgMCT4, however to a lesser extent than in BJ1-sgCTRL co-injections (**Supplementary Figure 8F**). Lastly, we determined MCT1 expression in HCC827+BJ1-sgCTRL tumors treated with metformin, to assess mitochondrial metabolism in carcinoma cells. Metformin decreased MCT1 expression in HCC827 cells, suggesting a reduction in mitochondrial metabolism due to OXPHOS inhibition by metformin (**Supplementary Figures 8G, H**). Altogether, these data suggest that the presence of MCT4-expressing fibroblasts induces mitochondrial metabolism in carcinoma cells, rendering them more susceptible to OXPHOS inhibition.

## Discussion

Tumors rely on the crosstalk between their different components in order to grow and disseminate. Our work contributes to the understanding of the metabolic interactions taking place between carcinoma cells and stromal CAFs in ADT cancers. Here, we have demonstrated that metabolic compartmentalization exists in NSCLC, as it had been previously described in HNSCC by our group (19). We have observed that the CAF-rich stromal compartment expresses high levels of MCT4 and low levels of IDH3α, markers of glycolysis, and that NSCLC carcinoma cells express high levels of the mitochondrial metabolism markers MCT1 and TOMM20. Moreover, we have begun to discern the mechanisms that drive this metabolic profile in ADT cancers. In co-culture models that recapitulate human metabolic compartmentalization, we have demonstrated that the presence of glycolytic CAFs promotes OXPHOS metabolism and ADT carcinoma cell aggressiveness, including increased proliferation and decreased apoptosis. Moreover, we have begun to define PEPCK-M as a possible marker of glycolytic reprogramming in fibroblasts. Also, in these ADT cancer models of tumor metabolic compartmentalization, we have demonstrated that targeting fibroblast metabolism may be an effective anti-tumor strategy. Pharmacological inhibition of HIF-1α and ROS, two main drivers of glycolysis in CAFs, partially revert the glycolytic phenotype by rescuing IDH3α and downregulating MCT4 expression in fibroblasts and reduce aggressiveness of ADT carcinoma cells. Finally, we have demonstrated that fibroblast MCT4 is a driver of carcinoma cell aggressiveness and tumor growth, and that it promotes mitochondrial metabolism in the carcinoma compartment, rendering tumors more susceptible to OXPHOS inhibitors.

Metabolic reprogramming of the tumor microenvironment and its role in supporting cancer growth is a promising research area for the development of novel anti-cancer agents. In 2009, Pavlides et al. proposed a new framework of cancer metabolism that took into consideration the role of the tumor stroma (59). In this model, reprogrammed CAFs adopt a glycolytic phenotype, upregulate MCT4 and secrete high energy metabolites such as lactate to the extracellular space, providing a nutrient-rich microenvironment to carcinoma cells. In turn, carcinoma cells upregulate the mitochondrial metabolism markers MCT1 and TOMM20, and use CAF-derived metabolites to obtain energy *via* OXPHOS (9–11). This metabolic cooperation, known as metabolic compartmentalization or symbiosis, has been confirmed in many human cancers, including HNSCC, and is associated with aggressive disease (13–25). Here, we show that markers of metabolic compartmentalization also exist in human NSCLC in a similar pattern as previously found in HNSCC (19). To our knowledge, this is the first study reporting that MCT4 is upregulated in the stroma of NSCLC, compared to the stroma of normal lung tissue. Consistent with previous reports, we also observed an increased expression of MCT1 in LUSC carcinoma cells, compared to normal pneumocytes, but not in LUAD (45, 60, 61). Even though in our cohort we found no MCT1 expression in LUAD samples, MCT1 has been found expressed at low levels in carcinoma cells in some LUAD patients (60). In fact, increased MCT1 expression has been associated with worse prognosis and poor survival both in LUSC and LUAD (44, 46, 60, 62, 63). Importantly, ours is the first study evaluating the expression of TOMM20 in human NSCLC and reporting that this marker of mitochondrial mass and OXPHOS is highly expressed in NSCLC compared to normal lung tissue. It is worth noting that in human HNSCC, MCT1 and TOMM20 are found highly expressed in the proliferative carcinoma cells (Ki-67 positive) of the leading edge which are the ones adjacent to the glycolytic tumor stroma expressing high MCT4 (19). Here, we did not assess carcinoma cell proliferation in human samples. However, this is the first instance where the combined expression of these three indicators of metabolic compartmentalization is studied in NSCLC, demonstrating the existence of high glycolysis in the stromal compartment and OXPHOS metabolism in the carcinoma compartment.

**Figure 9:**
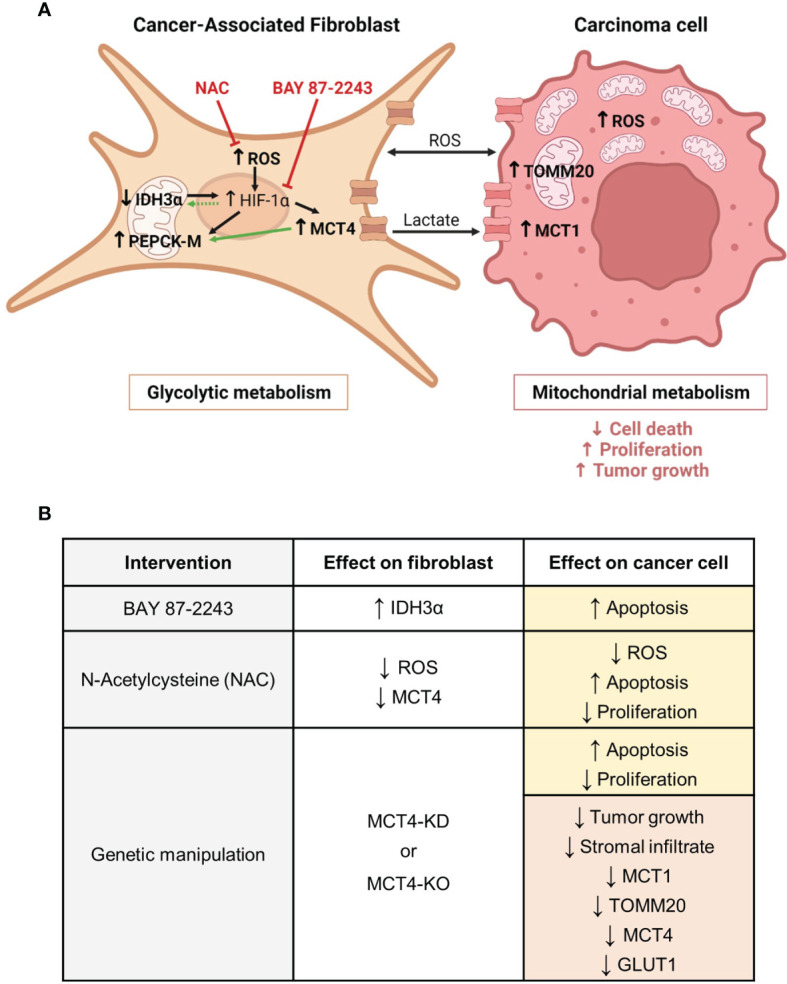
Metabolic compartmentalization in ADT cancers and interventions to modulate fibroblast metabolism and carcinoma cell aggressiveness. **(A)** We have demonstrated that both in patients and in experimental models of ADT cancers, CAFs are glycolytic, whereas carcinoma cells are mitochondria-rich and have OXPHOS metabolism. We have shown that CAFs have increased levels of ROS. ROS are a mean of communication between metabolic compartments and known inducers of HIF-1α stabilization in CAFs. HIF-1α drives glycolysis and transcriptionally regulates MCT4 and PEPCK-M. We have shown that CAFs have upregulation of MCT4 and PEPCK-M, and that PEPCK-M expression may also be regulated by MCT4. MCT4 is located in the plasma membrane and is the main exporter of glycolysis-derived lactate. Loss of IDH3α is also a driver of HIF-1α stabilization. We have shown that CAFs have downregulation of IDH3α expression and that elevated levels of HIF-1α may in turn maintain the low expression of IDH3α. Both upregulation of MCT4 and loss of IDH3α are markers of glycolysis in CAFs, and we define PEPCK-M as another possible marker of glycolytic reprogramming in CAFs. We have demonstrated that CAF glycolytic reprogramming can be targeted using a HIF-1α inhibitor (BAY 87-2243) and an antioxidant (NAC). In the carcinoma cell compartment, we have demonstrated that the presence of glycolytic CAFs induces ROS, MCT1 and TOMM20 expression and drives carcinoma cell aggressiveness, as seen by decreased cell death and increased proliferation, and tumor growth. In bold are the elements studied in our report. Black arrows indicate previously described pathways. Green arrows indicate the novel findings reported here. Red inhibitor arrows indicate NAC and BAY 87-2243 targets. **(B)** We have modulated CAF glycolytic metabolism and ADT carcinoma cell aggressiveness by means of pharmacological and genetic interventions. The HIF-1α inhibitor BAY 87-2243 rescued IDH3α expression in CAFs and induced carcinoma cell apoptosis. The antioxidant NAC reduced ROS levels and MCT4 expression in CAFs, and in carcinoma cells it reduced ROS levels, induced apoptosis and decreased proliferation. Genetic knock-down (KD) or knock-out (KO) of MCT4 in CAFs increased apoptosis and decreased proliferation in carcinoma cells, and reduced growth and the expression of MCT1, TOMM20, MCT4 and GLUT1 in tumors. Yellow boxes indicate effects *in vitro*. Red boxes indicate effects *in vivo*. CAF, Cancer-associated fibroblast; ROS, reactive oxygen species; HIF-1α, hypoxia inducible factor 1 alpha; MCT4, monocarboxylate transporter 4; IDH3α, isocitrate dehydrogenase 3 alpha; PEPCK-M, mitochondrial phosphoenolpyruvate carboxykinase; NAC, N-acetyl cysteine; MCT1, monocarboxylate transporter 1; TOMM20, translocase of the outer mitochondrial membrane 20. Created with BioRender.com.

CAFs, the main stromal cell type in ADT cancers, have pro-tumorigenic functions. Several studies have shown that reprogrammed CAFs enhance ADT carcinoma cell proliferation, invasion, metastasis, stemness and resistance to treatment in co-culture, conditioned media, and co-injection models (6, 64). Not surprisingly, the presence of CAFs is associated with advanced disease and poor survival in human ADT cancers (65–68). Consistent with others, we have shown that co-culturing ADT carcinoma cells with fibroblasts promote features of carcinoma cell aggressiveness, namely increased proliferation and decreased apoptosis (**Figure 9A**). Metabolic symbiosis with transfer of metabolites between carcinoma cells and CAFs *via* MCTs is an example of intercellular communication that promotes carcinoma cell progression (8, 55). Our co-culture models revealed drivers of metabolic symbiosis, with upregulation of MCT4 expression in BJ1 fibroblasts, and upregulation of TOMM20 in ADT carcinoma cells (**Figure 9A**). We also observed a downregulation of the TCA cycle enzyme IDH3α in co-cultured BJ1 (**Figure 9A**), which has been shown to induce glycolytic reprogramming in CAFs by stabilizing HIF-1α (33), the main regulator of glycolysis. Moreover, we observed for the first time that co-cultured BJ1 increased the expression of PEPCK-M (**Figure 9A**), a mitochondrial enzyme that regulates the metabolic adaptations in response to nutrient stress conditions in carcinoma cells (34, 36–38). One study has reported that PEPCK activity promotes glucose utilization and degradation to lactate in carcinoma cells, especially under glutamine restriction (36). Another study has shown that fibroblasts under glutamine deprivation upregulate the transcription factor ATF4 (69), which is an activator of PEPCK-M (70). Altogether, the data from the current study and others suggests that PEPCK-M is involved in the glycolytic reprogramming of CAFs. Interestingly, we found that in conditions that promote glycolysis in fibroblasts, namely high MCT4 expression and exposure to CSE (30, 51), PEPCK-M expression was upregulated. These results suggest that MCT4 and PEPCK-M are part of the same cluster of metabolic pathways, including glycolysis, that are reprogrammed upon co-culturing of fibroblasts with carcinoma cells. Glycolysis may regulate PEPCK-M expression and further studies will determine whether PEPCK-M can also modulate MCT4 expression and if it is necessary for the metabolic reprogramming of fibroblasts. Additionally, we confirmed that IDH3α is not expressed and that PEPCK-M is highly expressed in the tumor stroma of human NSCLC, and further studies will determine whether loss of IDH3α and/or high PEPCK-M may be used as glycolytic markers of CAFs and whether they could have any predictive value in ADT cancers. It is also worth noting that other studies using various carcinoma and fibroblast cell lines, including the ones used in our studies, have also demonstrated that the presence of fibroblasts promotes metabolic symbiosis in ADT cancers (13, 49, 50, 52, 71–75), indicating that metabolic compartmentalization may occur independently of the genetic background of carcinoma cells and the origin of fibroblasts.

Targeting the metabolism of the tumor stroma can potentially lead to a metabolic collapse in carcinoma cells. Here we have used two pharmacological strategies to modulate glycolysis in fibroblasts (**Figures 9A, B**). As previously mentioned, HIF-1α is a main driver of glycolysis as it transcriptionally activates many genes in the glycolytic pathway (9, 28, 29, 33). The HIF-1α inhibitor BAY 87-2243 upregulated the expression of IDH3α in BJ1 fibroblasts in co-culture with ADT carcinoma cells (**Figure 9B**), suggesting us that high HIF-1α expression keeps IDH3α expression at low levels to maintain the glycolytic phenotype in co-cultured fibroblasts. Further research will be needed to determine the existence of this feedback loop between HIF-1α and IDH3α. BAY 87-2243, however, did not change the expression of MCT4 in co-cultured BJ1 fibroblasts, even though MCT4 is transcriptionally activated by HIF-1α (29). This suggests that upregulation of MCT4 in co-cultured BJ1 fibroblasts may also be driven by other factors independently of HIF-1α activation, for example by ROS which has also been described to induce MCT4 in fibroblasts (9, 13, 22, 23, 51). Consistently, the antioxidant NAC decreased overall ROS levels in co-cultures of ADT carcinoma cells and BJ1 fibroblasts and decreased MCT4 expression specifically in BJ1 fibroblasts (**Figure 9B**). Similarly, NAC has been shown to decrease MCT4 in fibroblasts in co-culture with keratinocytes, breast and ovarian carcinoma cells, as well as in breast cancer patients (27, 76–78). HIF-1α has been shown to be necessary for the tumor-promoting effects of CAFs in NSCLC (79), and ROS are important signaling molecules that sustain, in part, the metabolic symbiosis between CAFs and carcinoma cells (58). Treatment with either BAY 87-2243 or NAC induced apoptosis and decreased proliferation in co-cultured ADT carcinoma cells (**Figure 9B**). To the best of our knowledge, we are the first to show that BAY 87-2243 and NAC can modulate glycolysis in fibroblasts and features of aggressiveness in carcinoma cells in models of ADT tumors. Further studies will be needed to determine whether this detrimental effect of BAY 87-2243 and NAC on carcinoma cell aggressiveness is at least partially mediated by the inhibition of glycolytic metabolism in fibroblasts.

Numerous elements support the importance of stromal MCT4 in ADT cancer progression. Elevated stromal MCT4 has been associated with decreased overall survival in HNSCC patients (32). In preclinical models, cigarette smoke, the main causing agent of ADT cancers, has been shown to induce glycolysis, MCT4 expression and lactate secretion in fibroblasts, and that these reprogrammed fibroblasts drive HNSCC tumor growth in an MCT4 dependent manner (51). Similarly, in a chemical carcinogenesis model using 4-nitroquinoline 1-oxide (4NQO), which mimics cigarette smoking, mice lacking MCT4 developed less invasive lesions in the tongue (80). The current study provides the first evidence that MCT4 expression in the stroma, and potentially in fibroblasts, is a key metabolic determinant of ADT cancer aggressiveness. We have shown that ADT carcinoma cells co-cultured with MCT4-knockdown (KD) BJ1 fibroblasts (BJ1-sgMCT4) and MCT4-KO MEFs have increased apoptotic rates and decreased proliferation compared to co-cultures with MCT4-expressing fibroblasts (BJ1-sgCTRL and WT-MEF) (**Figure 9B**). In various xenograft models, co-injections of ADT carcinoma cells with BJ1-sgMCT4 or MCT4-KO MEFs had a lower engraftment rate and generated smaller tumors than control co-injections (**Figure 9B**). Moreover, in two syngeneic models, co-injection of ADT carcinoma cells with MCT4-KO MEFs into MCT4-KO mice, where all the stromal infiltrate in tumors was MCT4-KO, generated smaller tumors than co-injection with WT MEFs in a WT background (**Figure 9B**). Interestingly, tumors generated from the co-injection of HCC827 with BJ1-sgCTRL fibroblasts had more stromal infiltrate, compared to BJ1-sgMCT4 co-injections (**Figure 9B**). Moreover, BJ1-sgCTRL fibroblasts persisted in tumors longer than BJ1-sgMCT4 fibroblasts and were enriched in the regions adjacent to carcinoma cell nests. The presence of an abundant stromal infiltrate could be promoting tumor growth and explain at least in part the differences in tumor growth between BJ1-sgCTRL and BJ1-sgMCT4 co-injections, as it is well established that the presence of fibroblasts enhances ADT cancer aggressiveness (6, 64–68). Changes in the tumor metabolism of different compartments are another possible mechanism to explain the differences in tumor growth. Co-injections of HCC827 with MCT4-KD and MCT4-KO fibroblasts had decreased expression of MCT4, GLUT1, MCT1 and TOMM20 in carcinoma cells (**Figure 9B**). MCT4, GLUT1 and MCT1 expression in carcinoma cells are prognostic biomarkers in ADT cancers (19, 39, 63, 81–86). TOMM20 has not been described as a prognostic biomarker in ADT cancers, however it is in other cancers (17, 47, 48). As previously mentioned, MCT1 and TOMM20 are drivers of OXPHOS in carcinoma cells used to define metabolic compartmentalization. Conversely, GLUT1 and MCT4 are markers of glucose uptake and glycolysis. It is worth noting that even though we have shown strong evidence of a stromal-cancer metabolic compartmentalization in human ADT tumors, we recognize that this is not the only metabolic profile found in these metabolically heterogeneous malignancies. Glycolytic carcinoma cells also exist in ADT cancers. However, markers of glycolysis are found on hypoxic carcinoma cells further away from the vasculature in ADT tumors, while markers of OXPHOS are found in well oxygenated carcinoma cells close to the stroma and blood vessels (84, 87, 88). Altogether, the data presented here support that ADT tumors are metabolically heterogeneous, and this promotes cancer aggressiveness. Moreover, it defines for the first time that fibroblast MCT4 is a contributor of carcinoma cell aggressiveness and a potential drug target in ADT cancers.

While there is still some disagreement on the predominant metabolism in ADT cancers, detailed studies in human subjects and in preclinical models demonstrate that NSCLC carcinoma cells rely on mitochondrial metabolism for proliferation and utilize lactate, rather than glucose, to fuel the TCA cycle (89–93). Moreover, experimental models show that lactate drives OXPHOS in ADT carcinoma cells, and that in turn lactate fuels their mitochondrial metabolism and promotes their growth (13, 49, 94–98). Altogether, this provides further evidence of the existence of a compartmentalized metabolism with high OXPHOS metabolism in ADT carcinoma cells. Consistent with this reliance on mitochondrial metabolism, here we have shown that tumors generated from the co-injection of HCC827 carcinoma cells and BJ1-sgCTRL fibroblasts, which had higher mitochondrial metabolism and less glucose dependence in the carcinoma compartment, were more susceptible to OXPHOS inhibition by metformin and phenformin, than tumors from co-injections with BJ1-sgMCT4. Moreover, in response to this OXPHOS inhibition, HCC827 carcinoma cells downregulated MCT1 expression and increased the uptake of glucose *in vivo*. Similarly, in co-culture conditions that promote metabolic compartmentalization, HCC827 carcinoma cells were more susceptible to OXPHOS inhibition by metformin and phenformin. Previous studies from our group had demonstrated that metformin affects carcinoma cell metabolism and inhibits tumor growth in HNSCC (74). Another study has shown that enhancing glycolysis in fibroblasts makes HNSCC carcinoma cells more sensitive to metformin (49). Altogether, the results from the current study and others suggest that metabolic compartmentalization with high MCT4 expression in CAFs and high mitochondrial metabolism in carcinoma cells renders carcinoma cells more susceptible to OXPHOS inhibition and reduces ADT tumor growth. Further studies will be needed to determine whether inhibition of OXPHOS in carcinoma cells decreases the ability to use stromal-derived mitochondrial substrates leading to the uncoupling of the metabolic symbiosis between carcinoma cells and CAFs, and whether this is a mechanism by which metformin and phenformin are able to decrease tumor growth in ADT cancers.

## Data Availability Statement

The original contributions presented in the study are included in the article/**Supplementary Material**. Further inquiries can be directed to the corresponding author.

## Ethics Statement

The animal study was reviewed and approved by Institutional Animal Care and Use Committee, Thomas Jefferson University.

## Author Contributions

MD-V and UM-O conceptualized and designed the study. MD-V developed the methodology. MD-V, DW-M, MM, ZL, MT, TZ contributed to the acquisition, analysis and interpretation of data. MD-V and UM-O contributed to the writing, review and/or revision of the manuscript. UM-O, NP, JJ and JC supervised the study. All authors contributed to the article and approved the submitted version.

## Funding

The National Cancer Institute of the National Institutes of Health under Award Numbers K08 CA175193 (UM-O), R37-CA234239 (UM-O) and 5P30CA056036-17 supported this work.

## Conflict of Interest

The authors declare that the research was conducted in the absence of any commercial or financial relationships that could be construed as a potential conflict of interest.

## Publisher’s Note

All claims expressed in this article are solely those of the authors and do not necessarily represent those of their affiliated organizations, or those of the publisher, the editors and the reviewers. Any product that may be evaluated in this article, or claim that may be made by its manufacturer, is not guaranteed or endorsed by the publisher.

## References

[1] Siegel RL Miller KD Fuchs HE Jemal A. Cancer Statistics, 2021. CA Cancer J Clin (2021) 71(1):7–33. doi: 10.3322/caac.21654 33433946

[2] Zappa C Mousa SA. Non-Small Cell Lung Cancer: Current Treatment and Future Advances. Transl Lung Cancer Res (2016) 5(3):288–300. doi: 10.21037/tlcr.2016.06.07 27413711 PMC4931124

[3] Johnson DE Burtness B Leemans CR Lui VWY Bauman JE Grandis JR. Head and Neck Squamous Cell Carcinoma. Nat Rev Dis Primers (2020) 6(1):92. doi: 10.1038/s41572-020-00224-3 33243986 PMC7944998

[4] Auperin A. Epidemiology of Head and Neck Cancers: An Update. Curr Opin Oncol (2020) 32(3):178–86. doi: 10.1097/CCO.0000000000000629 32209823

[5] Lu T Yang X Huang Y Zhao M Li M Ma K. Trends in the Incidence, Treatment, and Survival of Patients With Lung Cancer in the Last Four Decades. Cancer Manag Res (2019) 11:943–53. doi: 10.2147/CMAR.S187317 PMC6345192 30718965

[6] Zhang H Jiang H Zhu L Li J Ma S. Cancer-Associated Fibroblasts in Non-Small Cell Lung Cancer: Recent Advances and Future Perspectives. Cancer Lett (2021) 514:38–47. doi: 10.1016/j.canlet.2021.05.009 34019960

[7] Custodio M Biddle A Tavassoli M. Portrait of a CAF: The Story of Cancer-Associated Fibroblasts in Head and Neck Cancer. Oral Oncol (2020) 110:104972. doi: 10.1016/j.oraloncology.2020.104972 33011636

[8] Xing Y Zhao S Zhou BP Mi J. Metabolic Reprogramming of the Tumour Microenvironment. FEBS J (2015) 282(20):3892–8. doi: 10.1111/febs.13402 26255648

[9] Martinez-Outschoorn UE Lisanti MP Sotgia F. Catabolic Cancer-Associated Fibroblasts Transfer Energy and Biomass to Anabolic Cancer Cells, Fueling Tumor Growth. Semin Cancer Biol (2014) 25:47–60. doi: 10.1016/j.semcancer.2014.01.005 24486645

[10] Lisanti MP Martinez-Outschoorn UE Chiavarina B Pavlides S Whitaker-Menezes D Tsirigos A. Understanding the "Lethal" Drivers of Tumor-Stroma Co-Evolution: Emerging Role(s) for Hypoxia, Oxidative Stress and Autophagy/Mitophagy in the Tumor Micro-Environment. Cancer Biol Ther (2010) 10(6):537–42. doi: 10.4161/cbt.10.6.13370 PMC3040943 20861671

[11] Pavlides S Tsirigos A Migneco G Whitaker-Menezes D Chiavarina B Flomenberg N. The Autophagic Tumor Stroma Model of Cancer: Role of Oxidative Stress and Ketone Production in Fueling Tumor Cell Metabolism. Cell Cycle (2010) 9(17):3485–505. doi: 10.4161/cc.9.17.12721 PMC3047615 20861672

[12] Salem AF Whitaker-Menezes D Lin Z Martinez-Outschoorn UE Tanowitz HB Al-Zoubi MS. Two-Compartment Tumor Metabolism: Autophagy in the Tumor Microenvironment and Oxidative Mitochondrial Metabolism (OXPHOS) in Cancer Cells. Cell Cycle (2012) 11(13):2545–56. doi: 10.4161/cc.20920 PMC3404881 22722266

[13] Zhang Z Gao Z Rajthala S Sapkota D Dongre H Parajuli H. Metabolic Reprogramming of Normal Oral Fibroblasts Correlated With Increased Glycolytic Metabolism of Oral Squamous Cell Carcinoma and Precedes Their Activation Into Carcinoma Associated Fibroblasts. Cell Mol Life Sci (2020) 77(6):1115–33. doi: 10.1007/s00018-019-03209-y PMC11104868 31270582

[14] Curry JM Tassone P Cotzia P Sprandio J Luginbuhl A Cognetti DM. Multicompartment Metabolism in Papillary Thyroid Cancer. Laryngoscope (2016) 126(10):2410–8. doi: 10.1002/lary.25799 PMC4909595 26666958

[15] Knudsen ES Balaji U Freinkman E McCue P Witkiewicz AK. Unique Metabolic Features of Pancreatic Cancer Stroma: Relevance to the Tumor Compartment, Prognosis, and Invasive Potential. Oncotarget (2016) 7(48):78396–411. doi: 10.18632/oncotarget.11893 PMC5346648 27623078

[16] Afonso J Santos LL Morais A Amaro T Longatto-Filho A Baltazar F. Metabolic Coupling in Urothelial Bladder Cancer Compartments and its Correlation to Tumor Aggressiveness. Cell Cycle (2016) 15(3):368–80. doi: 10.1080/15384101.2015.1121329 PMC4943695 26636903

[17] Zhao Z Han F He Y Yang S Hua L Wu J. Stromal-Epithelial Metabolic Coupling in Gastric Cancer: Stromal MCT4 and Mitochondrial TOMM20 as Poor Prognostic Factors. Eur J Surg Oncol (2014) 40(10):1361–8. doi: 10.1016/j.ejso.2014.04.005 24821064

[18] Pertega-Gomes N Vizcaino JR Attig J Jurmeister S Lopes C Baltazar F. A Lactate Shuttle System Between Tumour and Stromal Cells is Associated With Poor Prognosis in Prostate Cancer. BMC Cancer (2014) 14:352. doi: 10.1186/1471-2407-14-352 24886074 PMC4039335

[19] Curry JM Tuluc M Whitaker-Menezes D Ames JA Anantharaman A Butera A. Cancer Metabolism, Stemness and Tumor Recurrence: MCT1 and MCT4 are Functional Biomarkers of Metabolic Symbiosis in Head and Neck Cancer. Cell Cycle (2013) 12(9):1371–84. doi: 10.4161/cc.24092 PMC3674065 23574725

[20] Martinez-Outschoorn UE Whitaker-Menezes D Valsecchi M Martinez-Cantarin MP Dulau-Florea A Gong J. Reverse Warburg Effect in a Patient With Aggressive B-Cell Lymphoma: Is Lactic Acidosis a Paraneoplastic Syndrome? Semin Oncol (2013) 40(4):403–18. doi: 10.1053/j.seminoncol.2013.04.016 23972703

[21] Fiaschi T Giannoni E Taddei ML Cirri P Marini A Pintus G. Carbonic Anhydrase IX From Cancer-Associated Fibroblasts Drives Epithelial-Mesenchymal Transition in Prostate Carcinoma Cells. Cell Cycle (2013) 12(11):1791–801. doi: 10.4161/cc.24902 PMC3713137 23656776

[22] Fiaschi T Marini A Giannoni E Taddei ML Gandellini P De Donatis A. Reciprocal Metabolic Reprogramming Through Lactate Shuttle Coordinately Influences Tumor-Stroma Interplay. Cancer Res (2012) 72(19):5130–40. doi: 10.1158/0008-5472.CAN-12-1949 22850421

[23] Whitaker-Menezes D Martinez-Outschoorn UE Lin Z Ertel A Flomenberg N Witkiewicz AK. Evidence for a Stromal-Epithelial “Lactate Shuttle” in Human Tumors: MCT4 is a Marker of Oxidative Stress in Cancer-Associated Fibroblasts. Cell Cycle (2011) 10(11):1772–83. doi: 10.4161/cc.10.11.15659 PMC3142461 21558814

[24] Martinez-Outschoorn UE Pavlides S Howell A Pestell RG Tanowitz HB Sotgia F. Stromal-Epithelial Metabolic Coupling in Cancer: Integrating Autophagy and Metabolism in the Tumor Microenvironment. Int J Biochem Cell Biol (2011) 43(7):1045–51. doi: 10.1016/j.biocel.2011.01.023 PMC3102770 21300172

[25] Migneco G Whitaker-Menezes D Chiavarina B Castello-Cros R Pavlides S Pestell RG. Glycolytic Cancer Associated Fibroblasts Promote Breast Cancer Tumor Growth, Without a Measurable Increase in Angiogenesis: Evidence for Stromal-Epithelial Metabolic Coupling. Cell Cycle (2010) 9(12):2412–22. doi: 10.4161/cc.9.12.11989 20562527

[26] Semenza GL. Regulation of Cancer Cell Metabolism by Hypoxia-Inducible Factor 1. Semin Cancer Biol (2009) 19(1):12–6. doi: 10.1016/j.semcancer.2008.11.009 19114105

[27] Martinez-Outschoorn UE Curry JM Ko YH Lin Z Tuluc M Cognetti D. Oncogenes and Inflammation Rewire Host Energy Metabolism in the Tumor Microenvironment: RAS and NFkappaB Target Stromal MCT4. Cell Cycle (2013) 12(16):2580–97. doi: 10.4161/cc.25510 PMC3865048 23860378

[28] Kierans SJ Taylor CT. Regulation of Glycolysis by the Hypoxia-Inducible Factor (HIF): Implications for Cellular Physiology. J Physiol (2021) 599(1):23–37. doi: 10.1113/JP280572 33006160

[29] Ullah MS Davies AJ Halestrap AP. The Plasma Membrane Lactate Transporter MCT4, But Not MCT1, is Up-Regulated by Hypoxia Through a HIF-1alpha-Dependent Mechanism. J Biol Chem (2006) 281(14):9030–7. doi: 10.1074/jbc.M511397200 16452478

[30] Tanner LB Goglia AG Wei MH Sehgal T Parsons LR Park JO. Four Key Steps Control Glycolytic Flux in Mammalian Cells. Cell Syst (2018) 7(1):49–62 e8. doi: 10.1016/j.cels.2018.06.003 29960885 PMC6062487

[31] Witkiewicz AK Whitaker-Menezes D Dasgupta A Philp NJ Lin Z Gandara R. Using the “Reverse Warburg Effect” to Identify High-Risk Breast Cancer Patients: Stromal MCT4 Predicts Poor Clinical Outcome in Triple-Negative Breast Cancers. Cell Cycle (2012) 11(6):1108–17. doi: 10.4161/cc.11.6.19530 PMC3335917 22313602

[32] Bovenzi CD Hamilton J Tassone P Johnson J Cognetti DM Luginbuhl A. Prognostic Indications of Elevated MCT4 and CD147 Across Cancer Types: A Meta-Analysis. BioMed Res Int (2015) 2015:242437. doi: 10.1155/2015/242437 26779534 PMC4686628

[33] Zhang D Wang Y Shi Z Liu J Sun P Hou X. Metabolic Reprogramming of Cancer-Associated Fibroblasts by IDH3alpha Downregulation. Cell Rep (2015) 10(8):1335–48. doi: 10.1016/j.celrep.2015.02.006 25732824

[34] Vincent EE Sergushichev A Griss T Gingras MC Samborska B Ntimbane T. Mitochondrial Phosphoenolpyruvate Carboxykinase Regulates Metabolic Adaptation and Enables Glucose-Independent Tumor Growth. Mol Cell (2015) 60(2):195–207. doi: 10.1016/j.molcel.2015.08.013 26474064

[35] Chun SY Johnson C Washburn JG Cruz-Correa MR Dang DT Dang LH. Oncogenic KRAS Modulates Mitochondrial Metabolism in Human Colon Cancer Cells by Inducing HIF-1alpha and HIF-2alpha Target Genes. Mol Cancer (2010) 9:293. doi: 10.1186/1476-4598-9-293 21073737 PMC2999617

[36] Montal ED Dewi R Bhalla K Ou L Hwang BJ Ropell AE. PEPCK Coordinates the Regulation of Central Carbon Metabolism to Promote Cancer Cell Growth. Mol Cell (2015) 60(4):571–83. doi: 10.1016/j.molcel.2015.09.025 PMC4656111 26481663

[37] Leithner K Hrzenjak A Trotzmuller M Moustafa T Kofeler HC Wohlkoenig C. PCK2 Activation Mediates an Adaptive Response to Glucose Depletion in Lung Cancer. Oncogene (2015) 34(8):1044–50. doi: 10.1038/onc.2014.47 24632615

[38] Hyrossova P Arago M Moreno-Felici J Fu X Mendez-Lucas A Garcia-Roves PM. PEPCK-M Recoups Tumor Cell Anabolic Potential in a PKC-Zeta-Dependent Manner. Cancer Metab (2021) 9(1):1. doi: 10.1186/s40170-020-00236-3 33413684 PMC7791766

[39] Leu M Kitz J Pilavakis Y Hakroush S Wolff HA Canis M. Monocarboxylate Transporter-1 (MCT1) Protein Expression in Head and Neck Cancer Affects Clinical Outcome. Sci Rep (2021) 11(1):4578. doi: 10.1038/s41598-021-84019-w 33633176 PMC7907348

[40] de Carvalho PA Bonatelli M Cordeiro MD Coelho RF Reis S Srougi M. MCT1 Expression is Independently Related to Shorter Cancer-Specific Survival in Clear Cell Renal Cell Carcinoma. Carcinogenesis (2021) 42(12):1420–7. doi: 10.1093/carcin/bgab100 34668521

[41] Payen VL Mina E Van Hee VF Porporato PE Sonveaux P. Monocarboxylate Transporters in Cancer. Mol Metab (2020) 33:48–66. doi: 10.1016/j.molmet.2019.07.006 31395464 PMC7056923

[42] Johnson JM Cotzia P Fratamico R Mikkilineni L Chen J Colombo D. MCT1 in Invasive Ductal Carcinoma: Monocarboxylate Metabolism and Aggressive Breast Cancer. Front Cell Dev Biol (2017) 5:27. doi: 10.3389/fcell.2017.00027 28421181 PMC5376582

[43] Latif A Chadwick AL Kitson SJ Gregson HJ Sivalingam VN Bolton J. Monocarboxylate Transporter 1 (MCT1) is an Independent Prognostic Biomarker in Endometrial Cancer. BMC Clin Pathol (2017) 17:27. doi: 10.1186/s12907-017-0067-7 29299023 PMC5745908

[44] Hong CS Graham NA Gu W Espindola Camacho C Mah V Maresh EL. MCT1 Modulates Cancer Cell Pyruvate Export and Growth of Tumors That Co-Express MCT1 and MCT4. Cell Rep (2016) 14(7):1590–601. doi: 10.1016/j.celrep.2016.01.057 PMC4816454 26876179

[45] Schuurbiers OC Meijer TW Kaanders JH Looijen-Salamon MG de Geus-Oei LF van der Drift MA. Glucose Metabolism in NSCLC is Histology-Specific and Diverges the Prognostic Potential of 18FDG-PET for Adenocarcinoma and Squamous Cell Carcinoma. J Thorac Oncol (2014) 9(10):1485–93. doi: 10.1097/JTO.0000000000000286 25170642

[46] Pinheiro C Longatto-Filho A Azevedo-Silva J Casal M Schmitt FC Baltazar F. Role of Monocarboxylate Transporters in Human Cancers: State of the Art. J Bioenerg Biomembr (2012) 44(1):127–39. doi: 10.1007/s10863-012-9428-1 22407107

[47] Roche ME Lin Z Whitaker-Menezes D Zhan T Szuhai K Bovee J. Translocase of the Outer Mitochondrial Membrane Complex Subunit 20 (TOMM20) Facilitates Cancer Aggressiveness and Therapeutic Resistance in Chondrosarcoma. Biochim Biophys Acta Mol Basis Dis (2020) 1866(12):165962. doi: 10.1016/j.bbadis.2020.165962 32920118 PMC7680391

[48] Park SH Lee AR Choi K Joung S Yoon JB Kim S. TOMM20 as a Potential Therapeutic Target of Colorectal Cancer. BMB Rep (2019) 52(12):712–7. doi: 10.5483/BMBRep.2019.52.12.249 PMC6941759 31818360

[49] Zhang X Dong Y Zhao M Ding L Yang X Jing Y. ITGB2-Mediated Metabolic Switch in CAFs Promotes OSCC Proliferation by Oxidation of NADH in Mitochondrial Oxidative Phosphorylation System. Theranostics (2020) 10(26):12044–59. doi: 10.7150/thno.47901 PMC7667693 33204328

[50] Wu X Zhou Z Xu S Liao C Chen X Li B. Extracellular Vesicle Packaged LMP1-Activated Fibroblasts Promote Tumor Progression *via* Autophagy and Stroma-Tumor Metabolism Coupling. Cancer Lett (2020) 478:93–106. doi: 10.1016/j.canlet.2020.03.004 32160975

[51] Domingo-Vidal M Whitaker-Menezes D Martos-Rus C Tassone P Snyder CM Tuluc M. Cigarette Smoke Induces Metabolic Reprogramming of the Tumor Stroma in Head and Neck Squamous Cell Carcinoma. Mol Cancer Res (2019) 17(9):1893–909. doi: 10.1158/1541-7786.MCR-18-1191 PMC6726526 31239287

[52] Wu J Hong Y Wu T Wang J Chen X Wang Z. Stromal-Epithelial Lactate Shuttle Induced by Tumorderived Interleukin1beta Promotes Cell Proliferation in Oral Squamous Cell Carcinoma. Int J Mol Med (2018) 41(2):687–96. doi: 10.3892/ijmm.2017.3267 PMC5752169 29207019

[53] Tuominen VJ Tolonen TT Isola J. ImmunoMembrane: A Publicly Available Web Application for Digital Image Analysis of HER2 Immunohistochemistry. Histopathology (2012) 60(5):758–67. doi: 10.1111/j.1365-2559.2011.04142.x 22296215

[54] Movafagh S Crook S Vo K. Regulation of Hypoxia-Inducible Factor-1a by Reactive Oxygen Species: New Developments in an Old Debate. J Cell Biochem (2015) 116(5):696–703. doi: 10.1002/jcb.25074 25546605

[55] Martinez-Outschoorn UE Peiris-Pages M Pestell RG Sotgia F Lisanti MP. Cancer Metabolism: A Therapeutic Perspective. Nat Rev Clin Oncol (2017) 14(2):113. doi: 10.1038/nrclinonc.2016.60 28094266

[56] Kapur A Felder M Fass L Kaur J Czarnecki A Rathi K. Modulation of Oxidative Stress and Subsequent Induction of Apoptosis and Endoplasmic Reticulum Stress Allows Citral to Decrease Cancer Cell Proliferation. Sci Rep (2016) 6:27530. doi: 10.1038/srep27530 27270209 PMC4897611

[57] Peiris-Pages M Martinez-Outschoorn UE Sotgia F Lisanti MP. Metastasis and Oxidative Stress: Are Antioxidants a Metabolic Driver of Progression? Cell Metab (2015) 22(6):956–8. doi: 10.1016/j.cmet.2015.11.008 26636492

[58] Martinez-Outschoorn UE Balliet RM Rivadeneira DB Chiavarina B Pavlides S Wang C. Oxidative Stress in Cancer Associated Fibroblasts Drives Tumor-Stroma Co-Evolution: A New Paradigm for Understanding Tumor Metabolism, the Field Effect and Genomic Instability in Cancer Cells. Cell Cycle (2010) 9(16):3256–76. doi: 10.4161/cc.9.16.12553 PMC3041164 20814239

[59] Pavlides S Whitaker-Menezes D Castello-Cros R Flomenberg N Witkiewicz AK Frank PG. The Reverse Warburg Effect: Aerobic Glycolysis in Cancer Associated Fibroblasts and the Tumor Stroma. Cell Cycle (2009) 8(23):3984–4001. doi: 10.4161/cc.8.23.10238 19923890

[60] Sanchez-Palencia A Gomez-Morales M Gomez-Capilla JA Pedraza V Boyero L Rosell R. Gene Expression Profiling Reveals Novel Biomarkers in Nonsmall Cell Lung Cancer. Int J Cancer (2011) 129(2):355–64. doi: 10.1002/ijc.25704 20878980

[61] Kuner R Muley T Meister M Ruschhaupt M Buness A Xu EC. Global Gene Expression Analysis Reveals Specific Patterns of Cell Junctions in non-Small Cell Lung Cancer Subtypes. Lung Cancer (2009) 63(1):32–8. doi: 10.1016/j.lungcan.2008.03.033 18486272

[62] Stewart PA Parapatics K Welsh EA Muller AC Cao H Fang B. A Pilot Proteogenomic Study With Data Integration Identifies MCT1 and GLUT1 as Prognostic Markers in Lung Adenocarcinoma. PloS One (2015) 10(11):e0142162. doi: 10.1371/journal.pone.0142162 26539827 PMC4634858

[63] Doherty JR Yang C Scott KE Cameron MD Fallahi M Li W. Blocking Lactate Export by Inhibiting the Myc Target MCT1 Disables Glycolysis and Glutathione Synthesis. Cancer Res (2014) 74(3):908–20. doi: 10.1158/0008-5472.CAN-13-2034 PMC3946415 24285728

[64] Wheeler SE Shi H Lin F Dasari S Bednash J Thorne S. Enhancement of Head and Neck Squamous Cell Carcinoma Proliferation, Invasion, and Metastasis by Tumor-Associated Fibroblasts in Preclinical Models. Head Neck (2014) 36(3):385–92. doi: 10.1002/hed.23312 PMC4111913 23728942

[65] Knops AM South A Rodeck U Martinez-Outschoorn U Harshyne LA Johnson J. Cancer-Associated Fibroblast Density, Prognostic Characteristics, and Recurrence in Head and Neck Squamous Cell Carcinoma: A Meta-Analysis. Front Oncol (2020) 10:565306. doi: 10.3389/fonc.2020.565306 33330034 PMC7729160

[66] Alcaraz J Carrasco JL Millares L Luis IC Fernandez-Porras FJ Martinez-Romero A. Stromal Markers of Activated Tumor Associated Fibroblasts Predict Poor Survival and are Associated With Necrosis in Non-Small Cell Lung Cancer. Lung Cancer (2019) 135:151–60. doi: 10.1016/j.lungcan.2019.07.020 31446988

[67] Karpathiou G Vieville M Gavid M Camy F Dumollard JM Magne N. Prognostic Significance of Tumor Budding, Tumor-Stroma Ratio, Cell Nests Size, and Stroma Type in Laryngeal and Pharyngeal Squamous Cell Carcinomas. Head Neck (2019) 41(6):1918–27. doi: 10.1002/hed.25629 30620425

[68] Shu H Li HF. Prognostic Effect of Stromal Myofibroblasts in Lung Adenocarcinoma. Neoplasma (2012) 59(6):658–61. doi: 10.4149/neo_2012_083 22862165

[69] Linares JF Cordes T Duran A Reina-Campos M Valencia T Ahn CS. ATF4-Induced Metabolic Reprograming Is a Synthetic Vulnerability of the P62-Deficient Tumor Stroma. Cell Metab (2017) 26(6):817–29 e6. doi: 10.1016/j.cmet.2017.09.001 28988820 PMC5718961

[70] Mendez-Lucas A Hyrossova P Novellasdemunt L Vinals F Perales JC. Mitochondrial Phosphoenolpyruvate Carboxykinase (PEPCK-M) is a Pro-Survival, Endoplasmic Reticulum (ER) Stress Response Gene Involved in Tumor Cell Adaptation to Nutrient Availability. J Biol Chem (2014) 289(32):22090–102. doi: 10.1074/jbc.M114.566927 PMC4139223 24973213

[71] Duda P Janczara J McCubrey JA Gizak A Rakus D. The Reverse Warburg Effect is Associated With Fbp2-Dependent Hif1alpha Regulation in Cancer Cells Stimulated by Fibroblasts. Cells (2020) 9(1):205. doi: 10.3390/cells9010205 PMC7016812 31947613

[72] Jiang E Xu Z Wang M Yan T Huang C Zhou X. Tumoral Microvesicle-Activated Glycometabolic Reprogramming in Fibroblasts Promotes the Progression of Oral Squamous Cell Carcinoma. FASEB J (2019) 33(4):5690–703. doi: 10.1096/fj.201802226R 30698991

[73] Cruz-Bermudez A Laza-Briviesca R Vicente-Blanco RJ Garcia-Grande A Coronado MJ Laine-Menendez S. Cancer-Associated Fibroblasts Modify Lung Cancer Metabolism Involving ROS and TGF-Beta Signaling. Free Radic Biol Med (2019) 130:163–73. doi: 10.1016/j.freeradbiomed.2018.10.450 30391585

[74] Tassone P Domingo-Vidal M Whitaker-Menezes D Lin Z Roche M Tuluc M. Metformin Effects on Metabolic Coupling and Tumor Growth in Oral Cavity Squamous Cell Carcinoma Coinjection Xenografts. Otolaryngol Head Neck Surg (2018) 158(5):867–77. doi: 10.1177/0194599817746934 29232177

[75] Luo M Luo Y Mao N Huang G Teng C Wang H. Cancer-Associated Fibroblasts Accelerate Malignant Progression of Non-Small Cell Lung Cancer *via* Connexin 43-Formed Unidirectional Gap Junctional Intercellular Communication. Cell Physiol Biochem (2018) 51(1):315–36. doi: 10.1159/000495232 30453281

[76] Monti D Sotgia F Whitaker-Menezes D Tuluc M Birbe R Berger A. Pilot Study Demonstrating Metabolic and Anti-Proliferative Effects of *In Vivo* Anti-Oxidant Supplementation With N-Acetylcysteine in Breast Cancer. Semin Oncol (2017) 44(3):226–32. doi: 10.1053/j.seminoncol.2017.10.001 PMC5737796 29248134

[77] Martinez-Outschoorn UE Balliet RM Lin Z Whitaker-Menezes D Howell A Sotgia F. Hereditary Ovarian Cancer and Two-Compartment Tumor Metabolism: Epithelial Loss of BRCA1 Induces Hydrogen Peroxide Production, Driving Oxidative Stress and NFkappaB Activation in the Tumor Stroma. Cell Cycle (2012) 11(22):4152–66. doi: 10.4161/cc.22226 PMC3524211 23047606

[78] Martinez-Outschoorn UE Balliet R Lin Z Whitaker-Menezes D Birbe RC Bombonati A. BRCA1 Mutations Drive Oxidative Stress and Glycolysis in the Tumor Microenvironment: Implications for Breast Cancer Prevention With Antioxidant Therapies. Cell Cycle (2012) 11(23):4402–13. doi: 10.4161/cc.22776 PMC3552923 23172369

[79] Zhang Y Bian Y Wang Y Wang Y Duan X Han Y. HIF-1alpha is Necessary for Activation and Tumour-Promotion Effect of Cancer-Associated Fibroblasts in Lung Cancer. J Cell Mol Med (2021) 25(12):5457–69. doi: 10.1111/jcmm.16556 PMC8184678 33943003

[80] Bisetto S Whitaker-Menezes D Wilski NA Tuluc M Curry J Zhan T. Monocarboxylate Transporter 4 (MCT4) Knockout Mice Have Attenuated 4nqo Induced Carcinogenesis; A Role for MCT4 in Driving Oral Squamous Cell Cancer. Front Oncol (2018) 8:324. doi: 10.3389/fonc.2018.00324 30211114 PMC6120975

[81] Fleming JC Woo J Moutasim K Mellone M Frampton SJ Mead A. HPV, Tumour Metabolism and Novel Target Identification in Head and Neck Squamous Cell Carcinoma. Br J Cancer (2019) 120(3):356–67. doi: 10.1038/s41416-018-0364-7 PMC6353968 30655616

[82] Tan Z Yang C Zhang X Zheng P Shen W. Expression of Glucose Transporter 1 and Prognosis in Non-Small Cell Lung Cancer: A Pooled Analysis of 1665 Patients. Oncotarget (2017) 8(37):60954–61. doi: 10.18632/oncotarget.17604 PMC5617397 28977837

[83] Eilertsen M Andersen S Al-Saad S Kiselev Y Donnem T Stenvold H. Monocarboxylate Transporters 1-4 in NSCLC: MCT1 is an Independent Prognostic Marker for Survival. PloS One (2014) 9(9):e105038. doi: 10.1371/journal.pone.0105038 25225794 PMC4165596

[84] Meijer TW Schuurbiers OC Kaanders JH Looijen-Salamon MG de Geus-Oei LF Verhagen AF. Differences in Metabolism Between Adeno- and Squamous Cell Non-Small Cell Lung Carcinomas: Spatial Distribution and Prognostic Value of GLUT1 and MCT4. Lung Cancer (2012) 76(3):316–23. doi: 10.1016/j.lungcan.2011.11.006 22153830

[85] Koukourakis MI Giatromanolaki A Bougioukas G Sivridis E. Lung Cancer: A Comparative Study of Metabolism Related Protein Expression in Cancer Cells and Tumor Associated Stroma. Cancer Biol Ther (2007) 6(9):1476–9. doi: 10.4161/cbt.6.9.4635 17881895

[86] Kunkel M Reichert TE Benz P Lehr HA Jeong JH Wieand S. Overexpression of Glut-1 and Increased Glucose Metabolism in Tumors are Associated With a Poor Prognosis in Patients With Oral Squamous Cell Carcinoma. Cancer (2003) 97(4):1015–24. doi: 10.1002/cncr.11159 12569601

[87] Stegeman H Rademakers SE Span PN Takes RP van der Kogel AJ Kaanders JH. Hypoxia, Metabolism, and Growth Factor Signaling in Head and Neck Squamous Cell Carcinoma: Correlation Between Primary and Xenograft Tumors. Head Neck (2014) 36(9):1288–95. doi: 10.1002/hed.23446 24668936

[88] Rademakers SE Lok J van der Kogel AJ Bussink J Kaanders JH. Metabolic Markers in Relation to Hypoxia; Staining Patterns and Colocalization of Pimonidazole, HIF-1alpha, CAIX, LDH-5, GLUT-1, MCT1 and MCT4. BMC Cancer (2011) 11:167. doi: 10.1186/1471-2407-11-167 21569415 PMC3115911

[89] Faubert B Li KY Cai L Hensley CT Kim J Zacharias LG. Lactate Metabolism in Human Lung Tumors. Cell (2017) 171(2):358–71.e9. doi: 10.1016/j.cell.2017.09.019 28985563 PMC5684706

[90] Hensley CT Faubert B Yuan Q Lev-Cohain N Jin E Kim J. Metabolic Heterogeneity in Human Lung Tumors. Cell (2016) 164(4):681–94. doi: 10.1016/j.cell.2015.12.034 PMC4752889 26853473

[91] Sellers K Fox MP Bousamra M 2nd Slone SP Higashi RM Miller DM. Pyruvate Carboxylase is Critical for non-Small-Cell Lung Cancer Proliferation. J Clin Invest (2015) 125(2):687–98. doi: 10.1172/JCI72873 PMC4319441 25607840

[92] Weinberg F Hamanaka R Wheaton WW Weinberg S Joseph J Lopez M. Mitochondrial Metabolism and ROS Generation are Essential for Kras-Mediated Tumorigenicity. Proc Natl Acad Sci U S A (2010) 107(19):8788–93. doi: 10.1073/pnas.1003428107 PMC2889315 20421486

[93] Fan TW Lane AN Higashi RM Farag MA Gao H Bousamra M. Altered Regulation of Metabolic Pathways in Human Lung Cancer Discerned by (13)C Stable Isotope-Resolved Metabolomics (SIRM). Mol Cancer (2009) 8:41. doi: 10.1186/1476-4598-8-41 19558692 PMC2717907

[94] Hu M Zhao Y Cao Y Tang Q Feng Z Ni J. DRP1 Promotes Lactate Utilization in KRAS-Mutant Non-Small-Cell Lung Cancer Cells. Cancer Sci (2020) 111(10):3588–99. doi: 10.1111/cas.14603 PMC7540982 32767829

[95] Romero-Garcia S Prado-Garcia H Valencia-Camargo AD Alvarez-Pulido A. Lactic Acidosis Promotes Mitochondrial Biogenesis in Lung Adenocarcinoma Cells, Supporting Proliferation Under Normoxia or Survival Under Hypoxia. Front Oncol (2019) 9:1053. doi: 10.3389/fonc.2019.01053 31681589 PMC6811519

[96] Wu H Ying M Hu X. Lactic Acidosis Switches Cancer Cells From Aerobic Glycolysis Back to Dominant Oxidative Phosphorylation. Oncotarget (2016) 7(26):40621–9. doi: 10.18632/oncotarget.9746 PMC5130031 27259254

[97] Chen YJ Mahieu NG Huang X Singh M Crawford PA Johnson SL. Lactate Metabolism is Associated With Mammalian Mitochondria. Nat Chem Biol (2016) 12(11):937–43. doi: 10.1038/nchembio.2172 PMC5069139 27618187

[98] Goodwin ML Jin H Straessler K Smith-Fry K Zhu JF Monument MJ. Modeling Alveolar Soft Part Sarcomagenesis in the Mouse: A Role for Lactate in the Tumor Microenvironment. Cancer Cell (2014) 26(6):851–62. doi: 10.1016/j.ccell.2014.10.003 PMC4327935 25453902

