# Supplementary material for: Monocarboxylate Transporter 4 in Cancer-Associated Fibroblasts Is a Driver of Aggressiveness in Aerodigestive Tract Cancers
Source: Front Oncol. 2022 Jun 22;12:906494. doi: 10.3389/fonc.2022.906494 (PMC9259095; doi:10.3389/fonc.2022.906494)
Supplement: Supplementary file 1 [file DataSheet_1.pdf]

Supplementary Material

Supplementary Methods

Immunohistochemistry quantification of human samples

Visual scoring and digital quantification by Aperio software were performed on 20 lung adenocarcinoma (LUAD) and 11 lung squamous cell carcinoma (LUSC) patient samples by two trained pathologists and two investigators. LUAD samples were visually scored. For stromal MCT4, several CAF-rich stromal areas in each sample were observed at 60X magnification. MCT4 staining was considered positive regardless of intensity. The percentage of positive stromal cells was crudely calculated in each area and the global percentage of MCT4-staining stromal cells was assigned accordingly. For MCT1 and TOMM20, several carcinoma cell areas in each slide were observed at 40X magnification. Percentage of cells with membranous (MCT1) or mitochondrial (TOMM20) staining as well as staining intensity were considered for the scoring. For all visual assessments, the percentage scores are presented as follows: 1+ (< 50%), 2+ (50%-90%), 3+ (> 90%). LUSC samples were scored by Aperio software (Aperio, Nussloch, Germany). Digital images were captured with Aperio slide scanner under 20X magnification and run in Aperio software under algorithms specific for membrane and mitochondrial staining patterns. Percentage scores were plotted.

TCGA Analyses

The R (http://www.R-project.org) was adopted to analyze TCGA database data for head and neck squamous cell cancer, lung adenocarcinoma and lung squamous cell cancer. Overall survival rates were compared with the log rank test for subjects with the highest and lowest quartile of expression of MCT4, MCT1 and TOMM20 and Kaplan Meier Curves were generated. Statistical significance was defined as p-values of less than 0.05.

Co-culture with inserts

A549, SCC9, SCC25 or BJ1 were seeded in 6 well plates in DMEM 10% HI-FBS. An insert with a 0.4 μm permeable PET membrane (Corning, 353090) was placed on the wells and BJ1, A549, H520 or SCC25 were seeded on top of the insert in DMEM 10% HI-FBS. After 24 hours, media was changed to DMEM 10% NuSerum. Co-cultures were allowed to grow for 3 days. The inserts were then removed and protein lysates were obtained from the cells at the bottom of the well for immunoblotting.

Sorter

Co-cultures of A549 and BJ1-GFP were seeded in 10 cm dishes at a total number of 12 x 10^5 cells/dish in DMEM 10% HI-FBS. After 24 hours, media was changed to DMEM 10% Nu-FBS and co-cultures were grown for 3 days. A549 and BJ1-GFP were separated by Fluorescence-activated cell sorting (FACS) based on GFP signal. Protein lysate from BJ1-GFP fibroblasts was obtained for immunoblotting.

Conditioned media generation

Co-cultures of A549, HCC827, H226, H520, SCC9, and SCC25 with BJ1 fibroblasts were seeded in 60 mm dishes at a total number of 4 x 10^5 cells per dish. Co-cultures were grown for 3 days in DMEM

10% Nu-FBS. Then, co-culture conditioned media (CM) was collected and spun down (2,000 rpm for 10 min) to remove cells and debris and diluted in DMEM 10% Nu-FBS to generate 50% CM.

Fibroblast treatment with CM

BJ1 fibroblasts were plated in 6-well plates at a confluence of 1 x 10^5 cells/well in DMEM 10% HI-FBS. After 24 hours, 50% CM from co-cultures was added to BJ1 fibroblasts. Cells were cultured in conditioned media for 3 days. Protein lysates of BJ1 fibroblasts were obtained for immunoblotting.

Cigarette Smoke Extract

Cigarette smoke extract and exposure to BJ1 fibroblasts and MEFs was performed as previously described (51).

Immunoblotting

Protein extraction, quantification and immunoblotting were performed as described previously (51). Primary antibodies used were anti-MCT4 (Alomone labs, AMT-014), anti-LDH-V (Abcam, 101562), anti-IDH3α (A-10, Santa Cruz, sc-398021), anti-PEPCK-M (Abcam, ab70359), anti-TOMM20 (F-10, Santa Cruz, sc-17764), anti-β-actin (Sigma-Aldrich, A5441), and anti-β-tubulin (Sigma-Aldrich, T4026).

Transwell migration assay

12–well polycarbonate plates with 12-mm diameter inserts and 8-mm pore–sized membranes (Costar, 353182) were used to study the migratory capabilities of carcinoma cells in response to fibroblasts. 2.5 x 10^4 A549 or 3.75 x 10^4 HCC827 or SCC25 were seeded on top of the insert membrane, and 12.5 x 10^4 or 11.25 x 10^4 BJ1 were seeded at the bottom of the well in DMEM 10% HI-FBS. The following day, media was changed to DMEM 10% Nu-serum. Carcinoma cells were allow to migrate for 24 hours. Membranes were fixed with 70% ethanol for 15 minutes, stained in 0.5% crystal violet for 15 minutes, and rinsed with water. Images of the membranes were taken under an inverted microscope and the number of migrated cells per field was counted. At least 4 fields were counted per membrane.

Cisplatin treatment

Co-cultures of A549 or HCC827 and BJ1 fibroblasts were treated with 5 µM cisplatin (Sigma, 232120) for 48 hours prior to PFBF assessment by flow cytometry.

Stromal quantification with ImageJ

Images of whole tumors were taken at 4X magnification. Areas of stroma were selected with the ImageJ freehand tool. The percentage of area from the whole tumor comprised by stroma was quantified.

In-vivo Imaging System (IVIS)

Mice were anesthetized with inhaled isoflurane (2%) and placed in an In-vivo Imaging System (Perkin Elmer). Fluorescence from mCherry-tagged BJ1-sgCTRL and BJ1-sgMCT4 fibroblasts was acquired in auto-exposure with 550/650 excitation/emission filters in IVIS and signal intensity was quantified in ImageJ. An identical region of interest (ROI) was drawn around the fluorescent signal for each tumor. The “threshold” tool was used to select the fluorescent signal. The same threshold value was

used for all tumors for comparison. The integrated density of the fluorescent signal was measured in pixels.

Metformin and phenformin treatments in vitro and in vivo

Monocultures and co-cultures of cancer cells and BJ1 fibroblasts were treated for 48 hours with 200 μM of metformin or 150 μM of phenformin diluted into the growth media or left untreated in regular growth media as controls.

Metformin or phenformin were administered by the drinking water to mice bearing HCC827+BJ1-sgCTRL/BJ1-sgMCT4 tumors. Treatment with 5 mg/ml metformin water was started at day 4post-tumor injection. Metformin water was changed every 3 days for the duration of the experiment (40 days). Treatment with phenformin was started 10 days post-tumor injection and maintained for the duration of the experiment (26 days). Phenformin was administered orally in the drinking water at 1 mg/ml and 1.7 mg/ml for the first 10 days, and subsequently administered intraperitoneally diluted in PBS at 50 mg/kg and 100 mg/kg for the last 6 days. All treated mice received the same dosage of phenformin. 5% sucrose was added to phenformin water to increase palatability. Untreated mice were given 5% sucrose water or injected with PBS alone.

2-NBDG assessment in xenografts

Mice were injected intraperitoneally with 100 μl of 20 mM 2-NBDG (ThermoFisher, N13195). After 2 hours, mice were euthanized, and tumors were collected and dissociated as previously described (protocol from Lukacs RU, 2010, Nat. Protocols). Each tumor was placed in a 10 cm dish containing 1 mL of dissecting media (DMEM 10% HI-FBS 1X PenStrep) and minced using a razor blade. Minced tumor tissue was transferred to a 15 mL Falcon tube containing 9 mL of dissecting media. 1 mL of 10X Collagenase solution (Sigma-Aldrich, SCR103) was added (final concentration of collagenase was 1 mg/ml). Tumor tissue in collagenase solution was incubated in a 37°C bath for 2 hours, shaking every 15 minutes, then collagenase solution was removed by centrifugation (1300 rpm for 5 min). The pellet of minced tissue was resuspended in 2 mL of warm trypsin/0.05% EDTA and incubated for 5 min at 37°C. A P1000 pipette was used to pipette the tissue up and down in the trypsin solution, then 2 mL of dissecting media were added. The solution containing minced tumor tissue was passed through a 18G syringe 5 times, and then through a 20G syringe 5 times. The resulting solution was then filtered through a nylon mesh filter with a 40 μm pore size. Cells mixed with trypan blue were counted with a hemocytometer, and 3-5 x 10^5 viable cells were transferred to a flow cytometry tube. Cells were spun down to remove trypsin solution and then stained with anti-human EpCAM-Alexa Fluor 647 (Biolegend, 324212) in PBS for 20 min at 4°C. Cells were spun down and resuspended in PBS for flow cytometry.

Supplementary Figures

Suppl. Figure 1

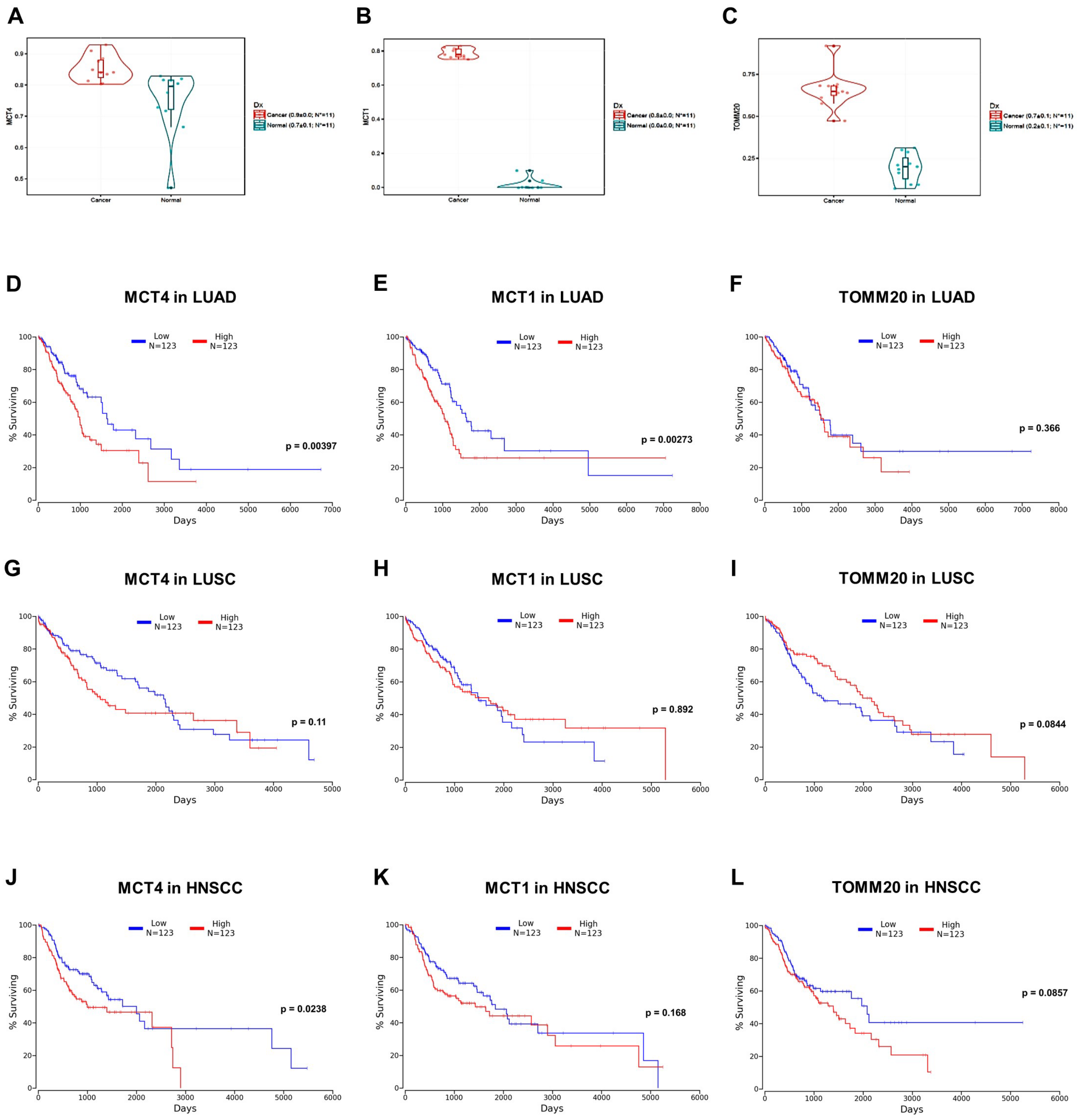

Supplementary Figure 1. Quantification of metabolic compartmentalization markers in human lung squamous cell carcinoma (LUSC). Aperio digital pathology interpretation was used for quantification of immunohistochemistry staining. **(A)** Quantification of stromal MCT4, **(B)** quantification of cancer cell MCT1, and **(C)** quantification of cancer cell TOMM20 in LUSC tissue (in red) and in adjacent normal lung (in blue). Percentage scores were assessed using membrane and co-localization algorithms. Student's t-test was used for statistical analyses (MCT4 $p=0.007$, MCT1 $p<0.001$, TOMM20 $p<0.001$). **(D-L)** Kaplan Meier Curves for MCT4, MCT1 and TOMM20 expression in LUAD, LUSC and HNSCC. Log rank test was used for statistical analyses (significance at $p < 0.05$).

Suppl. Figure 2

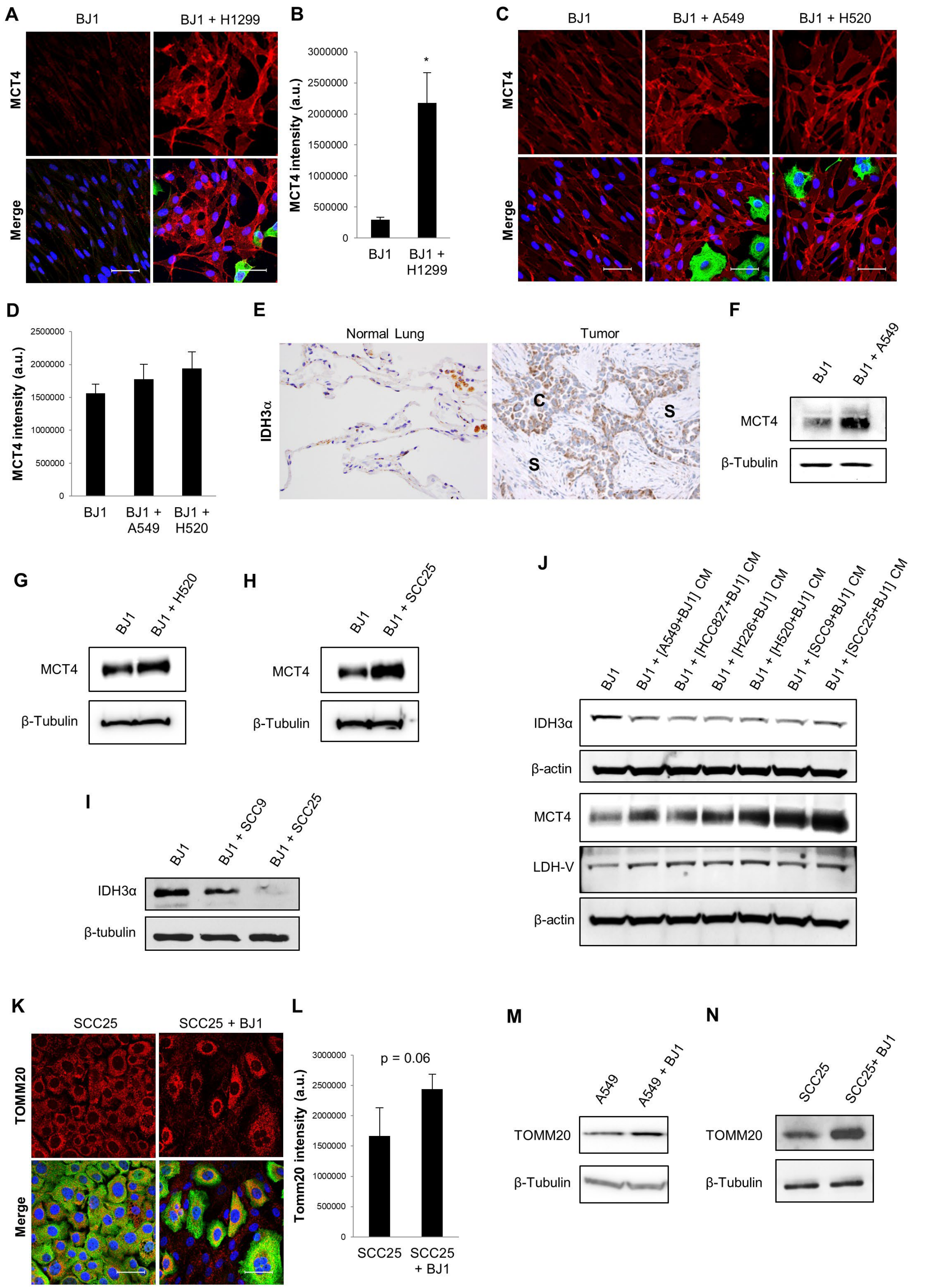

Supplementary Figure 2. Expression of markers of metabolic compartmentalization in co-cultures of ADT carcinoma cells and fibroblasts. Human ADT carcinoma cell lines H1299, A549, H520, SCC9 and SCC25 were co-cultured with BJ1 fibroblasts for 4 days. Monocultures of carcinoma cells and BJ1 fibroblasts were maintained in parallel as controls. **(A)** Confocal imaging and **(B)** quantification of MCT4 expression in BJ1 fibroblasts in monoculture or in co-culture with H1299. **(C)** Confocal imaging and **(D)** quantification of MCT4 expression in BJ1 fibroblasts in monoculture or in co-culture with A549 and H520. **(E)** IDH3α expression in human normal lung and in NSCLC. Images were taken at 20X from tumor tissue and adjacent normal lung tissue within the same sample (C, cancer; S, stroma). **(F-H)** BJ1 were co-cultured with A549, H520 or SCC25 carcinoma cells through a transwell membrane or maintained alone. MCT4 expression by western blot in BJ1 monocultured or co-cultured with A549 **(F)**, H520 **(G)**, and SCC25 **(H)**. **(I)** IDH3α expression by western blot in BJ1 in monoculture or in co-culture with SCC9 and SCC25. **(J)** IDH3α, MCT4 and LDH-V expression by western blot of BJ1 cultured with standard media or with 50% conditioned media generated from co-cultures of ADT carcinoma cells with BJ1. **(K)** Confocal imaging and **(L)** quantification of TOMM20 expression in SCC25 in monoculture or in co-culture with BJ1. **(M, N)** A549 or SCC25 carcinoma cells were monocultured of co-cultured with BJ1 through a transwell membrane. TOMM20 expression by western blot in A549 **(M)** and SCC25 **(N)**. For all confocal images (panels A, C and K), MCT4 and TOMM20 staining are shown in red, cancer cells are shown in green (K8/18 staining), and nuclei are shown in blue (DAPI). MCT4 and TOMM20 staining were quantified using ImageJ. Confocal microscopy images were acquired at the 40X magnification. Student's t-test was used for statistical analyses (* $p<0.05$). (a.u., arbitrary units. Scale bar = 50 μm)

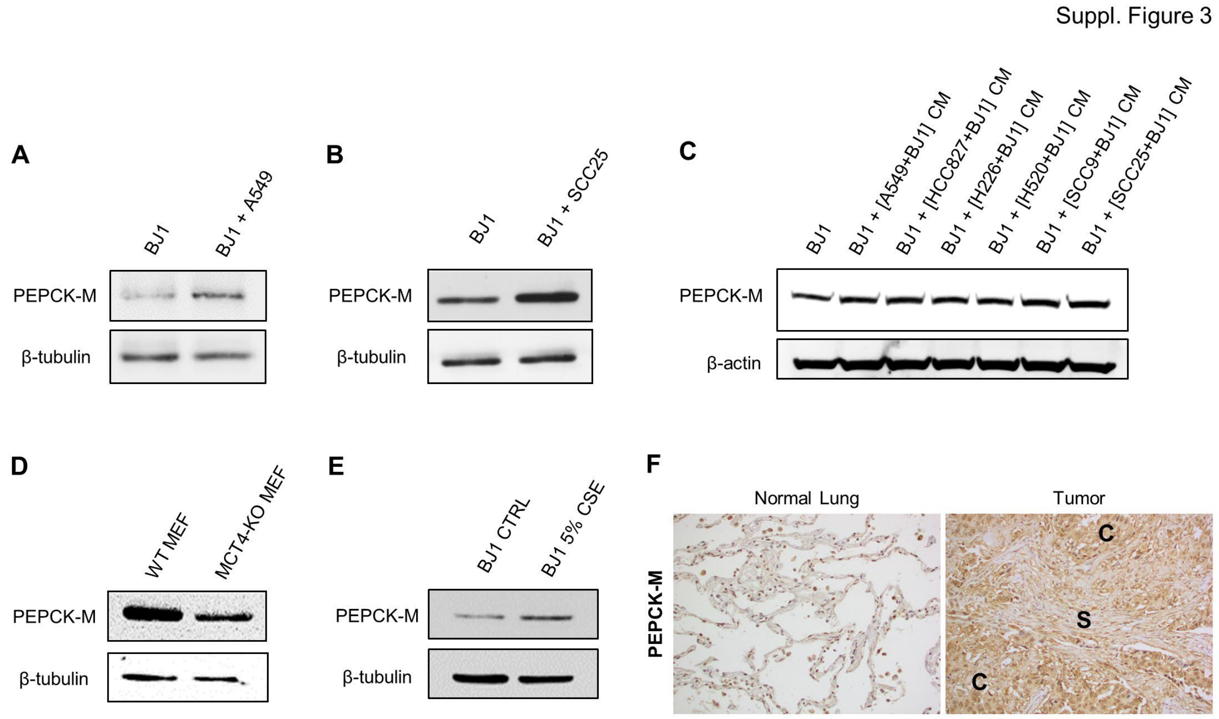

Supplementary Figure 3. PEPCK-M expression in glycolytic fibroblasts. Western blot assessments of PEPCK-M expression in fibroblasts under different conditions inducing glycolysis. **(A, B)** PEPCK-M expression in BJ1 fibroblasts in monoculture or in co-culture with A549 **(A)** and SCC25 **(B)**. **(C)** PEPCK-M expression in BJ1 cultured with standard media or with 50% conditioned media generated from co-cultures of ADT carcinoma cells with BJ1. **(D)** PEPCK-M expression in WT and MCT4-KO MEFs. **(E)** PEPCK-M expression in control BJ1 and in BJ1 exposed to 5% cigarette smoke extract (CSE). **(F)** PEPCK-M expression in human normal lung and in NSCLC. Images were taken at 20X from tumor tissue and adjacent normal lung tissue within the same sample (C, cancer; S, stroma).

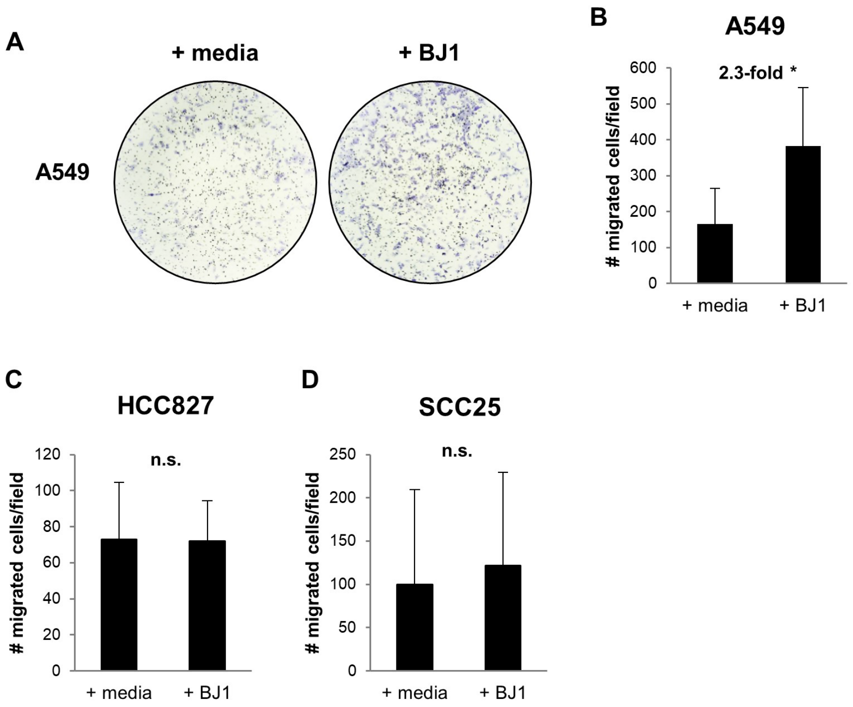

Supplementary Figure 4. Effects of fibroblasts on carcinoma cell migration. **(A-D)** A549, HCC827 and SCC25 carcinoma cells were seeded on top of a transwell membrane and regular growth media or media containing BJ1 were seeded at the bottom of the well. **(A, B)** Crystal violet staining **(A)** and quantification of number of A549 cells migrated through the membrane **(B)**. **(C, D)** Quantification of number of HCC827 **(C)** and SCC25 **(D)** cells migrated through the membrane. Student's t-test was used for statistical analyses (* $p<0.05$).

Suppl. Figure 5

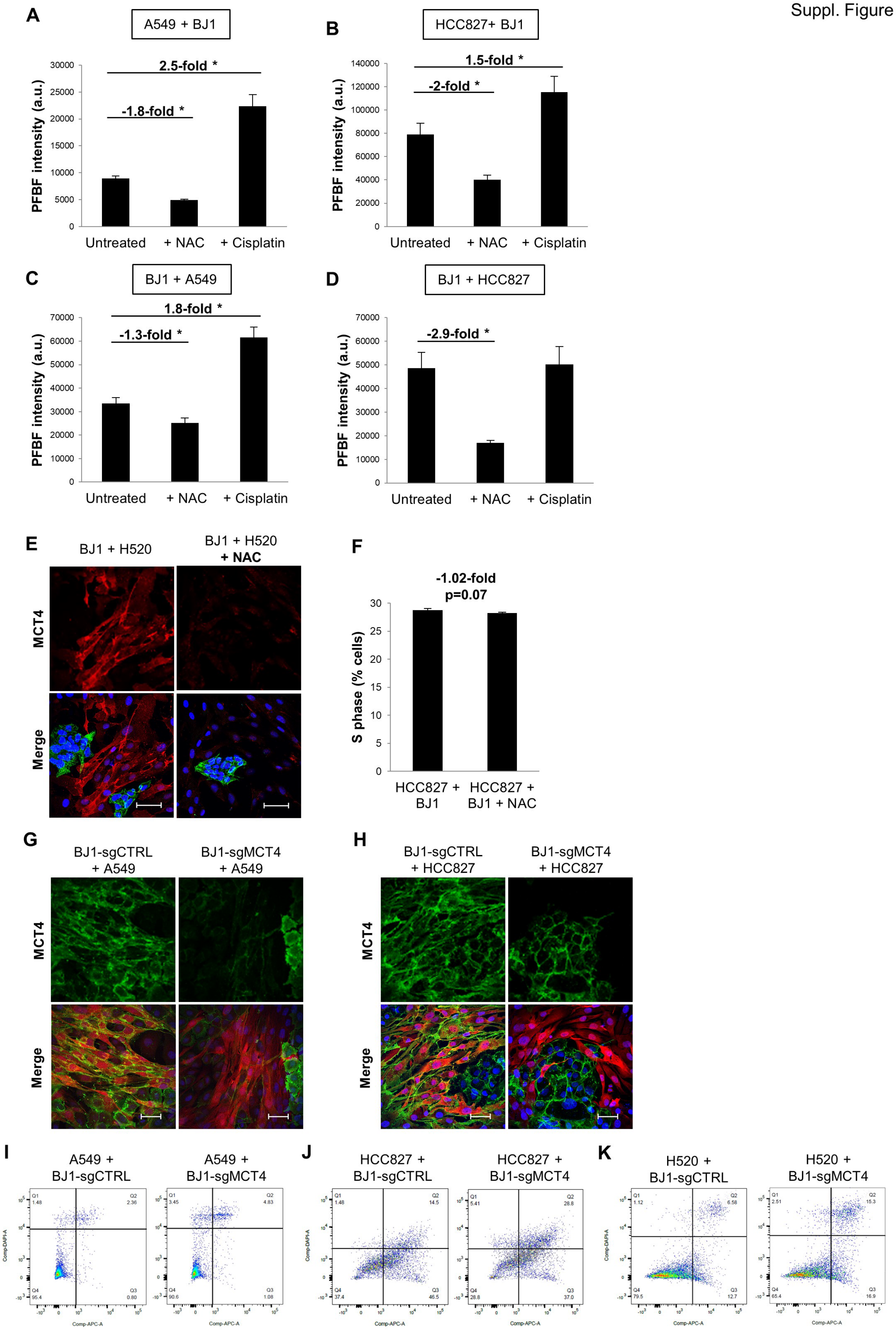

Supplementary Figure 5. Effects of ROS neutralization and MCT4 downregulation on fibroblast metabolism and ADT cancer cell aggressiveness. **(A, B)** Hydrogen peroxide (H_2O_2) levels measured by flow cytometric analysis of the PFBF probe in A549 **(A)** and HCC827 **(B)** co-cultured with BJ1, untreated or treated with N-acetyl cysteine (NAC) or cisplatin. (**C, D**) H_2O_2 levels in BJ1 co-cultured with A549 **(C)** and HCC827 **(D)**, untreated or treated with NAC or cisplatin. **(E)** MCT4 expression by confocal imaging in BJ1 co-cultured with H520 untreated or treated with NAC. MCT4 staining is shown in red, cancer cells are shown in green (K8/18 staining), and nuclei are shown in blue (DAPI). **(F)** Percentage of HCC827 cells incorporating EdU (S phase) in co-culture with BJ1, untreated or treated with NAC. **(G, H)** MCT4 expression by confocal imaging in co-cultures of A549 **(G)** and HCC827 **(H)** with mCherry-tagged BJ1-sgCTRL or BJ1-sgMCT4. MCT4 is shown in green, BJ1 are shown in red (mCherry), and nuclei are shown in blue (DAPI). Confocal microscopy images were acquired at the 40X magnification. **(I-K)** Flow cytometry plots with gating strategy for apoptosis and cell death assessment in A549 **(I)**, HCC827 **(J)** and H520 **(K)** co-cultured with BJ1-sgCTRL or BJ1-sgMCT4. Student's t-test was used for statistical analyses (* $p<0.05$). (a.u., arbitrary units. Scale bar = 50 μm)

Suppl. Figure 6

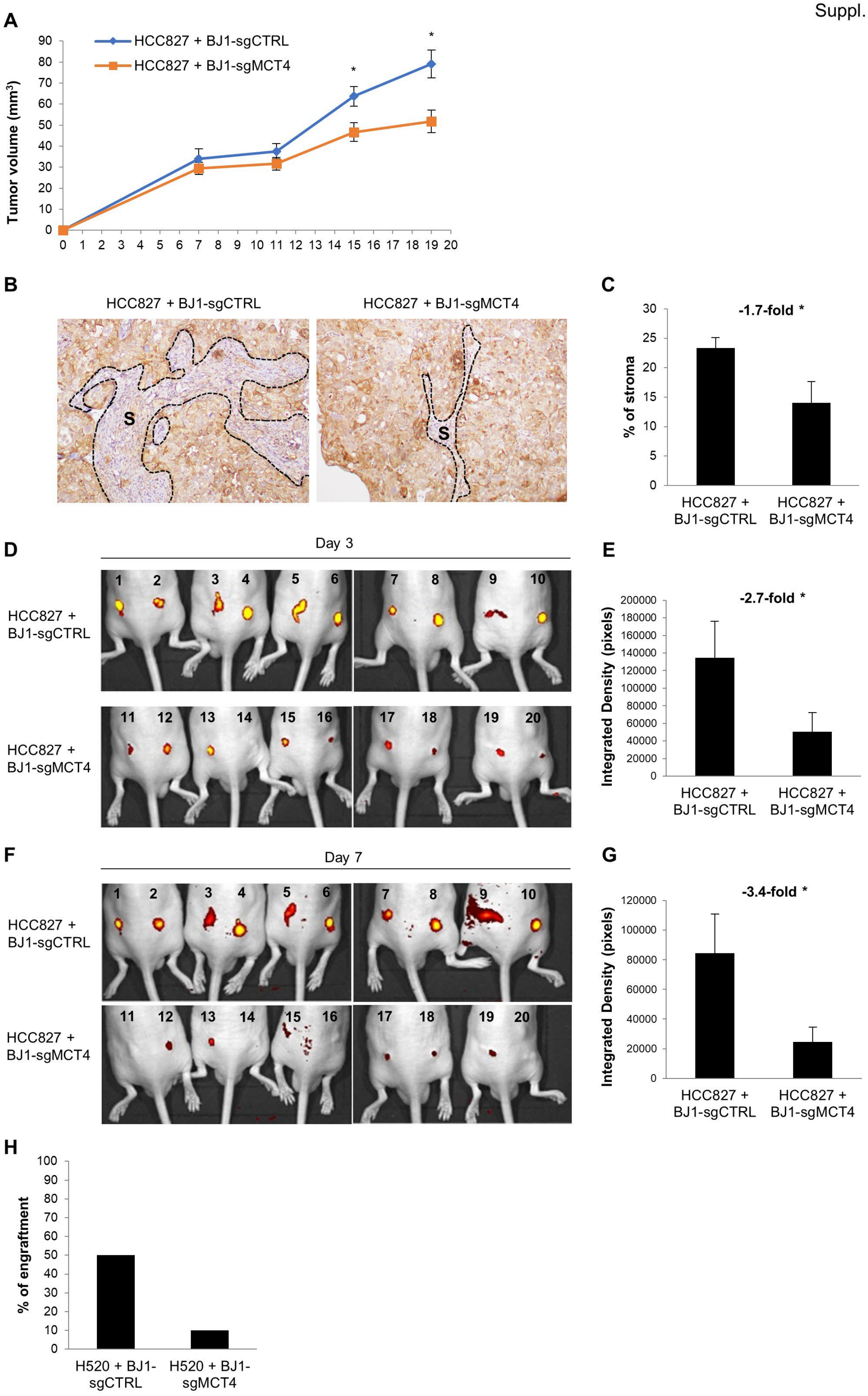

Supplementary Figure 6. Effects of fibroblast MCT4 on tumor growth. **(A)** Tumor growth overtime of HCC827 co-injected with BJ1-sgCTRL or BJ1-sgMCT4 into nude mice. **(B)** Representative images of HCC827+BJ1-sgCTRL and HCC827+BJ1-sgMCT4 tumors showing areas of high stromal infiltration and **(C)** percentage of the whole tumor parenchyma comprised by stroma. Images were acquired at 10X magnification (S, Stroma). **(D-G)** Detection of mCherry signal from injected BJ1-sgCTRL and BJ1-sgMCT4 with an In Vivo Imaging System (IVIS) and quantification of detected signal by ImageJ. Images were acquired at days 3 **(D, E)** and 7 **(F, G)** post-tumor implantation. Note that in panel E, tumor number 9 at day 7 showed high levels of background signal and was excluded from analysis. **(H)** Percentage of tumor engraftment from co-injections of H520 with BJ1-sgCTRL or BJ1-sgMCT4 into nude mice. Student's t-test was used for statistical analyses (* $p<0.05$).

Suppl. Figure 7

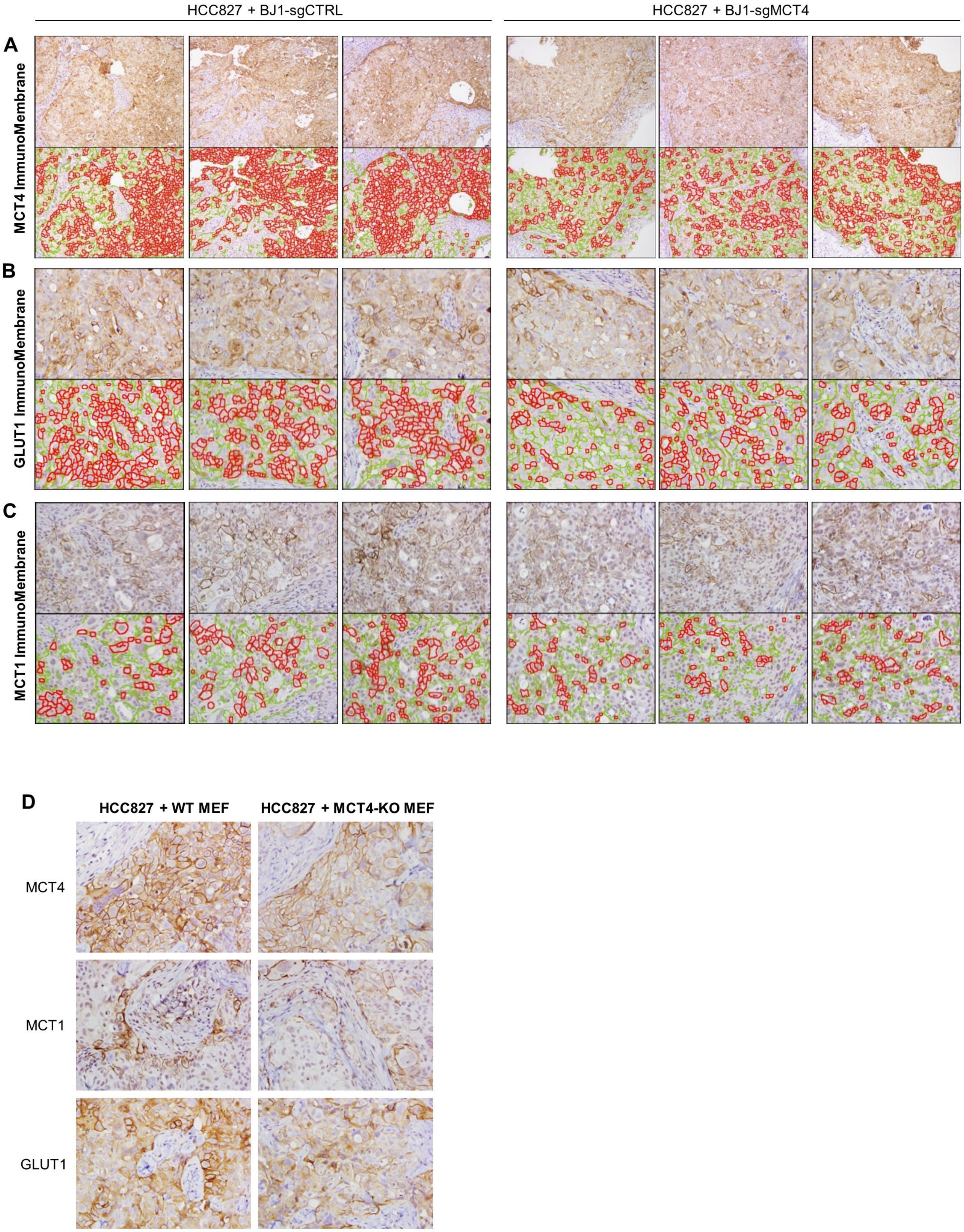

Supplementary Figure 7. Effects of fibroblast MCT4 on tumor metabolism. **(A-C)** ImmunoMembrane analysis of MCT4 **(A)**, GLUT1 **(B)** and MCT1 **(C)** staining in tumors generated from the co-injection of HCC827 with BJ1-sgCTRL or BJ1-sgMCT4 in nude mice. ImmunoMembrane software labels in red the strong/complete membranous staining and in green the weak or incomplete membranous staining. Images were taken at 20X. **(D)** Immunohistochemical assessment of MCT4, MCT1 and GLUT1 expression in tumors generated from the co-injection of HCC827 and WT or MCT4-KO MEF in nude mice. Images were taken at 40X.

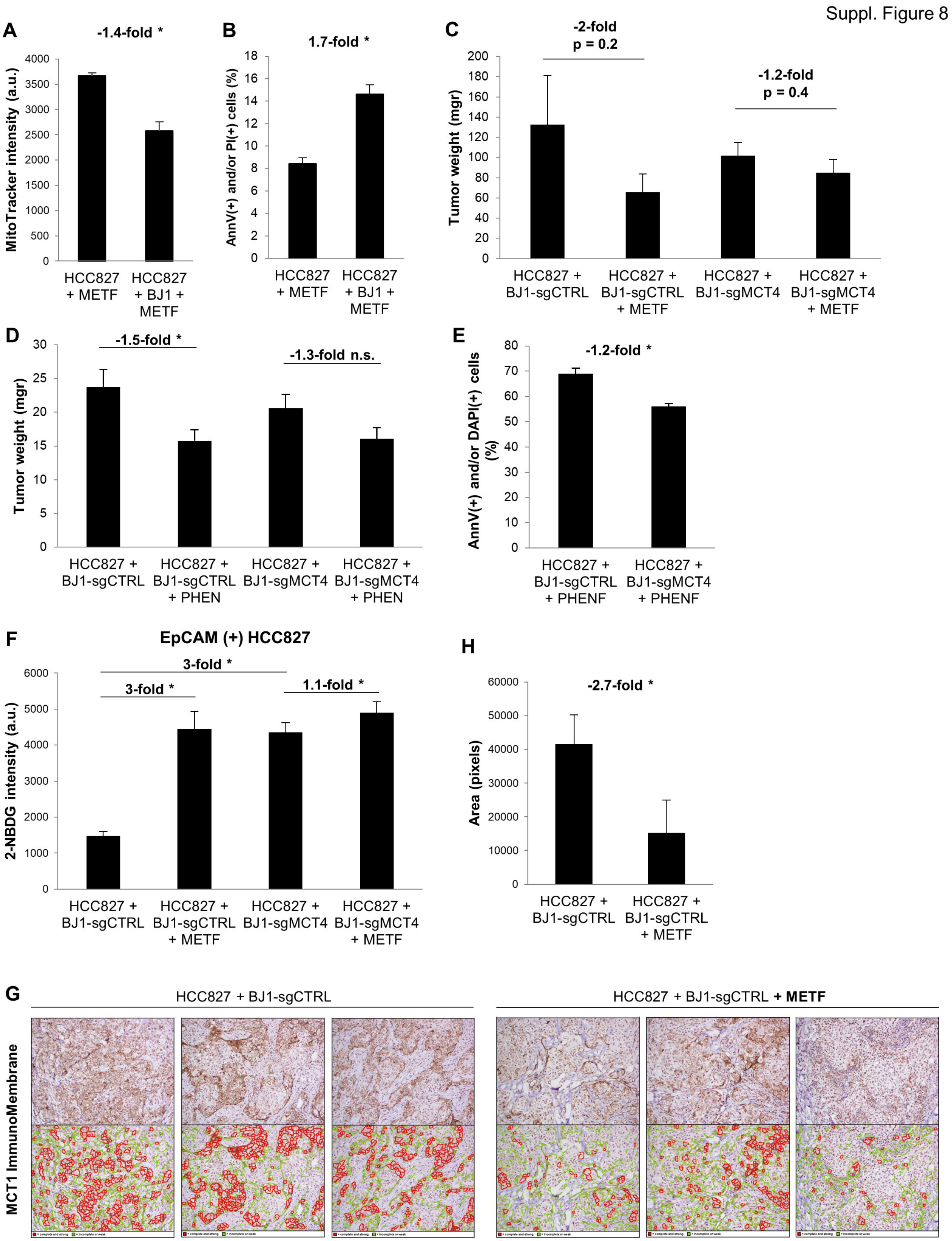
Suppl. Figure 8
A
-1.4-fold *
MitoTracker intensity (a.u.)
HCC827 + METF
HCC827 + BJ1 + METF
B
1.7-fold *
AnnV(+) and/or PI(+) cells (%)
HCC827 + METF
HCC827 + BJ1 + METF
C
-2-fold p = 0.2
-1.2-fold p = 0.4
Tumor weight (mgr)
HCC827 + BJ1-sgCTRL
HCC827 + BJ1-sgCTRL + METF
HCC827 + BJ1-sgMCT4
HCC827 + BJ1-sgMCT4 + METF
D
-1.5-fold *
-1.3-fold n.s.
Tumor weight (mgr)
HCC827 + BJ1-sgCTRL
HCC827 + BJ1-sgCTRL + PHEN
HCC827 + BJ1-sgMCT4
HCC827 + BJ1-sgMCT4 + PHEN
E
-1.2-fold *
AnnV(+) and/or DAPI(+) cells (%)
HCC827 + BJ1-sgCTRL + PHENF
HCC827 + BJ1-sgMCT4 + PHENF
F
EpCAM (+) HCC827
3-fold *
3-fold *
1.1-fold *
2-NBDG intensity (a.u.)
HCC827 + BJ1-sgCTRL
HCC827 + BJ1-sgCTRL + METF
HCC827 + BJ1-sgMCT4
HCC827 + BJ1-sgMCT4 + METF
H
-2.7-fold *
Area (pixels)
HCC827 + BJ1-sgCTRL
HCC827 + BJ1-sgCTRL + METF
G
HCC827 + BJ1-sgCTRL
HCC827 + BJ1-sgCTRL + METF
MCT1 ImmunoMembrane

Supplementary Figure 8. OXPHOS inhibition decreases cancer cell aggressiveness and tumor growth. **(A)** Flow cytometry assessment of mitochondrial membrane potential by MitoTracker in HCC827 in monoculture or in co-culture with BJ1 treated with metformin (METF). **(B)** Flow cytometry assessment of apoptosis (AnnV staining) and cell death (PI staining) in HCC827 in monoculture or in co-culture with BJ1 treated with METF. **(C, D)** Tumor weight of co-injections of HCC827 with BJ1-sgCTRL or BJ1-sgMCT4 into nude mice untreated or treated with METF **(C)** and phenformin (PHEN) **(D)**. **(E)** Flow cytometry assessment of apoptosis and cell death (DAPI staining) in HCC827 co-cultured with BJ1-sgCTRL or BJ1-sgMCT4 untreated or treated with PHEN. **(F)** Flow cytometry assessment of 2-NBDG (glucose analogue) uptake *in vivo* in HCC827 cells co-injected with BJ1-sgCTRL or BJ1-sgMCT4, into nude mice untreated or treated with METF. Anti-human EpCAM antibody staining was used to detect the injected HCC827 cells from disaggregated xenograft tissue. **(G)** ImmunoMembrane analysis of MCT1 expression in HCC827 co-injected with BJ1-sgCTRL into mice untreated or treated with METF. ImmunoMembrane software labels in red the strong/complete membranous staining and in green the weak or incomplete membranous staining. **(H)** ImageJ quantification of the red labeling from ImmunoMembrane software analysis. Images were taken at 20X. Student's t-test was used for statistical analyses (* $p<0.05$). (a.u., arbitrary units)

Supplementary Tables

Score:	**0+ (0%)**	**1+ (<50%)**	**2+ (50%-90%)**	**3+ (>90%)**
**MCT4**				
Normal lung fibroblasts	20	0	0	0
Tumor stroma CAFs	0	4	13	3
**MCT1**				
Normal lung pneumocytes	20	0	0	0
Lung ADC cancer cells	20	0	0	0
**TOMM20**				
Normal lung pneumocytes	20	0	0	0
Lung ADC cancer cells	0	4	7	8

Supplementary Table 1. Scoring of MCT4, MCT1 and TOMM20 immunohistochemical staining in human lung adenocarcinoma (LUAD) tissue and adjacent normal lung tissue. MCT4 was scored in fibroblasts within normal lung tissue and in cancer-associated fibroblasts (CAFs) within the tumor stroma. MCT1 and TOMM20 were scored in pneumocytes within the normal lung tissue and in cancer cells within the tumor parenchyma. Samples were scored based on the percentage of positive staining cells in 0+, 1+, 2+ and 3+.
